# Sub-inhibitory concentrations of oxacillin modulate biogenesis and function of extracellular vesicles secreted by oxacillin-sensitive methicillin-resistant *Staphylococcus aureus*

**DOI:** 10.3389/fmicb.2025.1616536

**Published:** 2025-08-04

**Authors:** Zhaoxia Ran, Minghui Hao, Binxin Guo, Junrui Wang

**Affiliations:** ^1^Department of Laboratory Medicine, Affiliated Hospital of Inner Mongolia Medical University, Hohhot, China; ^2^Medical Experimental Center, General Hospital of Ningxia Medical University, Yinchuan, Ningxia, China; ^3^Inner Mongolia Key Laboratory of Clinical Pathogenic Microorganism, The Affiliated Hospital of Inner Mongolia Medical University, Hohhot, China

**Keywords:** OS-MRSA, extracellular vesicles, sub-inhibitory concentrations, oxacillin, bioactivities

## Abstract

**Background:**

Extracellular vesicles (EVs) derived from *Staphylococcus aureus* (*S. aureus*) carry multiple components, such as toxins, antigens, and resistance determinants, whose production is influenced by exposure to β-lactam antibiotics. However, systematic studies on the effects of β-lactam antibiotics on the release and functions of EVs remain limited.

**Methods:**

Extracellular vesicles (EVs) were isolated from the OS-MRSA (OS-200) strain by ultracentrifugation, including both EVs untreated with antibiotics and those exposed to sub-inhibitory concentrations of oxacillin (OI-EVs). Proteomic analysis was performed using liquid chromatography-tandem mass spectrometry (LC-MS/MS). The functional changes of these EVs were further assessed through erythrocyte hemolysis assays, biofilm formation assays, measurement of cytokines, and cell invasion assays.

**Results:**

Exposure to sub-inhibitory concentrations of oxacillin (half and one-eighth of the minimum inhibitory concentration [MIC]) significantly enhanced the secretion of OI-EVs and increased the abundance of several proteins, including penicillin-binding protein 2, EmrB, Hlg, and enolase, in a concentration-dependent manner. Notably, EV1/2MIC exhibited more pronounced functional changes and enhanced hemolytic activity against rabbit red blood cells. Also, EV1/2MIC exhibited significant promoting effects on biofilm formation of OS-200. Additionally, OI-EVs stimulated the secretion of interleukin-6 and tumor necrosis factor-α by THP-1 macrophages in a dose-dependent manner and demonstrated greater penetration potential into A549 lung epithelial cells.

**Conclusion:**

Exposure to sub-inhibitory concentrations of oxacillin significantly altered the secretion and composition of EVs, highlighting a novel relationship between antimicrobial exposure, exosome biogenesis, and OS-MRSA pathogenicity. These findings provide new insights into how β-lactam antibiotics influence host–pathogen interactions by modulating the secretion of bacterial vesicles.

## 1 Introduction

*Staphylococcus aureus* is a prominent pathogenic Gram-positive bacterium that can cause a wide range of human infections, from superficial skin infections to systemic infections (Lowy, 1998). The emergence and prevalence of multidrug resistant and methicillin-resistant *S. aureus* (MRSA) clones promoted this pathogen to the status of a superbug (Lakhundi and Zhang, 2018). To maintain the progress of infection, *S. aureus* produces extracellular vesicles (SaEVs) that deliver virulence factors or antimicrobial resistance-related biomaterials to host cells (Liu et al., 2023). The biogenesis and secretion of SaEVs are highly complex processes, and the proteins encapsulated within SaEVs exhibit pronounced heterogeneity. They differ substantially across various strains and production conditions, which in turn leads to functional diversity (Jeon et al., 2016; Briaud et al., 2021; Wang et al., 2021). For example, phenol-soluble modulins function at the membrane level, promoting vesicle budding from the cytoplasmic membrane, whereas the porosity of the cell wall is regulated by peptidoglycan crosslinking and autolysin production (Wang et al., 2018).

The secretion of SaEVs is influenced by numerous factors, including temperature, oxidative stress, iron limitation, and sub-inhibitory concentrations of ethanol and antibiotics (Wang et al., 2021). *β*-lactam antibiotics, especially, affect the secretion of SaEVs prominently. By binding to penicillin-binding proteins, β-lactams can prevent cell wall synthesis, causing cell lysis and death (Lim and Strynadka, 2002). SaEVs contain a *β*-lactamase protein, BlaZ, that can hydrolyze β-lactam antibiotics, thereby shielding *S. aureus* from antibiotics (Lee et al., 2013). Moreover, exposure to ampicillin affects the secretion of SaEVs and further influences bacterial antibiotic resistance by altering the protein composition of the vesicles (Kim et al., 2020). However, few studies have explored the effects of virulence regulation in *S. aureus* mediated by altered SaEVs exposed to *β*-lactam antibiotics (Liu et al., 2023).

Oxacillin-sensitive MRSA (OS-MRSA), a distinct subgroup of MRSA, is characterized by the presence of the *mecA* gene; however, despite its genotypic potential for resistance, it remains phenotypically susceptible to oxacillin (Song et al., 2017). Studies have indicated that the detection rate of OS-MRSA is often underestimated in routine clinical practice owing to the limitations of conventional phenotypic detection methods, such as agar diffusion and broth microdilution (Duarte et al., 2024). In addition, guidelines issued by the European Committee on Antimicrobial Susceptibility Testing also recommend routine laboratories to incorporate both phenotypic and molecular diagnostic approaches to enhance the accuracy of OS-MRSA detection. Sub-inhibitory concentrations of oxacillin may act as an inducer, promoting the growth of subpopulations with high levels of resistance to oxacillin, which may lead to more serious consequences (Park et al., 2024). A previous study demonstrated that exposing OS-MRSA to the *β*-lactam antibiotic ceftazidime altered the bioactivity and underlying mechanisms of EVs produced by the bacteria (He et al., 2019). However, no study has examined how oxacillin affects the production of EVs from OS-MRSA and further mediates their activities.

This study aimed to explore the effects of sub-inhibitory concentrations of oxacillin on the EVs produced by OS-MRSA and changes in their functions, including their hemolytic activity, biofilm-forming activity, cytotoxicity, and *in vitro* immune activation capability.

## 2 Materials and methods

### 2.1 Isolation and identification of *S. aureus* strain OS-200

The *S. aureus* strain, OS-200, used in this study was identified and analyzed in a previous study (Liu et al., 2021), where it was isolated from the bronchoalveolar lavage fluid of an 8-year-old child admitted to the Affiliated Hospital of Inner Mongolian Medical University in 2016. To validate the identity of the strain, the isolate was reidentified during this study using matrix-assisted laser desorption/ionization time-of-flight mass spectrometry performed on the Zybio EXS3000 system (Zybio Inc., China). The analysis confirmed the strain to be *S. aureus* (Supplementary File 1). Phenotypic susceptibility testing was performed using the broth microdilution method following the guidelines stated in Clinical Laboratory and Standards Institute M100-S32 (Clinical and Laboratory Standards Institute, 2022; Humphries et al., 2021). Heterogeneous resistance was detected using population analysis profiling approach (Liu et al., 2021). These results verified the identity of OS-200 as a classical OS-MRSA strain-genotypically resistant (due to *mecA*), phenotypically susceptible to oxacillin (minimum inhibitory concentration [MIC] = 1 μg/mL), but harboring low-frequency oxacillin-resistant subpopulations.

### 2.2 Isolation and purification of EVs

This study featured one control group (no oxacillin) and two experimental groups-one supplemented with oxacillin at one-eighth the MIC (EV_1/8MIC_ group) and the other supplemented with oxacillin at half the MIC (EV_1/2MIC_ group). EVs were isolated and purified as previously described, with a few alterations (Su et al., 2022). Briefly, the OS-200 strain was cultured in tryptic soy broth (TSB) with or without oxacillin at 37°C under shaking conditions for 24 h. Bacterial cultures were centrifuged at 7,000 × *g* for 15 min, and the resultant supernatants were filtered through a membrane with a pore size of 0.22 μm (Merck Millipore, Tullagreen, Carrigtwohill, Co. Cork, Ireland) to remove residual bacteria and debris. Subsequently, the filtrate was concentrated 10-fold under nitrogen pressure using a 100-kDa ultrafiltration membrane (Amicon®, Jaffrey, NH, USA) equipped with a magnetic stirrer. The concentrated supernatants were ultracentrifuged at 100,000 × *g* for 4 h at 4°C. Subsequently, the EVs pellet was resuspended in PBS and subjected to an additional centrifugation step for 1 h. The resulting EVs pellets were gently resuspended in PBS, and the solution was sterilized by filtration through a 0.22-μm membrane to obtain the EVs preparation used in this study.

### 2.3 Transmission electron microscopy

Purified EVs were adsorbed onto 300-mesh Formvar/carbon-coated copper grids (Electron Microscopy Sciences, Hatfield, PA, USA) for 1 min at room temperature. Excess liquid was removed by blotting with a filter paper. The samples were subjected to negative staining with 1% uranyl acetate for 10 s, air-dried, and observed under a JEOL 1200EX transmission electron microscope (JEOL, Peabody, MA, USA) operated at 80 kV and equipped with an AMT 2 k CCD camera (Advanced Microscopy Techniques Corp, Danvers, MA). Images were acquired at magnifications ranging from 20,000 × to 100,000×, with representative micrographs at scale bars of 100 nm and 500 nm selected for final analysis (Corona et al., 2023).

### 2.4 Nanoparticle tracking analysis

Nanoparticle tracking analysis was performed using a ZetaView PMX 110 Particle Tracking Analyzer (Particle Metrix, Meerbusch, Germany). The isolated EVs were diluted in 1 × PBS (VivaCell, Shanghai, China) to achieve a particle concentration within the optimal detection range of 1 × 10^6^–1 × 10^7^ particles/ml. Prior to measurement, the instrument was calibrated using 100-nm polystyrene latex beads (ThermoFisher Scientific, USA). The diluted EVs samples were analyzed at 11 different positions in the measurement cell to ensure representative sampling. All measurements were conducted at a temperature between 23°C and 30°C. The dilution factor used for each sample was recorded and applied during data analysis to accurately calculate particle concentration (Longjohn and Christian, 2022).

### 2.5 Protein extraction and quantification of EVs

EVs were isolated from 400 mL of bacterial culture with an approximate cell density of 1 × 10^9^ colony forming units/ml, yielding approximately 1 × 10^10^ EV particles. For protein extraction, EV pellets were lysed in buffer containing 8 M urea (GibcoBRL), 30 mM HEPES (pH 8.5), 1 mM phenylmethylsulfonyl fluoride (AMRECSO), 2 mM EDTA (AMRECSO), and 10 mM dithiothreitol (Promega). The lysates were ultrasonicated in an ice bath for 5 min (pulse on 2 s, off 3 s, 180 W) and centrifuged at 20,000 × *g* for 30 min at 4°C. To the soluble protein fraction, dithiothreitol was added to a final concentration of 10 Mm. The samples were incubated at 56°C for 1 h, alkylated with 55 mM iodoacetamide (Promega) for 1 h in the dark at room temperature, and centrifuged at 20,000 × *g* for 30 min at 4°C. Finally, the supernatants were collected, and protein concentrations were determined using a commercial Bradford Protein Assay Kit (Amesco) following the manufacturer’s instructions (Lee et al., 2009).

### 2.6 Sodium dodecyl sulfate–polyacrylamide gel electrophoresis

EVs isolated from the three groups were analyzed using sodium dodecyl sulfate–polyacrylamide gel electrophoresis. Each sample was diluted with a 5 × loading buffer containing sodium dodecyl sulfate (Sigma-Aldrich, USA), dithiothreitol (Sigma-Aldrich, USA), phenylmethylsulfonyl fluoride (Beyotime Biotechnology, China), and EDTA (Amresco, USA). Protein denaturation was achieved by heating the samples at 95°C for 5 min in a THRMS dry bath incubator (Ningbo Xinzhi Biotechnology Co., China). Subsequently, either 10 μg of protein or 20 μL of the prepared protein mixture was loaded onto 12% self-prepared polyacrylamide resolving gels layered with 5% stacking gels. A prestained protein molecular weight marker (14–170 kDa; Thermo Fisher Scientific, USA) was used as a reference standard. Electrophoresis was performed using the PAC200 vertical electrophoresis system (Bio-Rad, USA) under a constant voltage of 120 V until the bromophenol blue dye front reached the bottom of the gel. After electrophoresis, the gels were stained with BPI-Blue high-sensitivity protein stain (Beijing Proteome Institute, China) for 1 h at room temperature, destained using a proprietary destaining solution until the background was clear and the protein bands were sharply resolved, and imaged at a resolution of 400 pixels per inch using a UMAX Powerlook 2,100 scanner (UMAX Technologies, Taiwan). The images were processed and saved using Powerlook 2100 software (UMAX Technologies, Taiwan) (Brennan et al., 2020).

### 2.7 Proteomic analysis using liquid chromatography–tandem mass spectrometry

The isolated EVs were lysed and subjected to a modified trypsin digestion protocol prior to liquid chromatography–tandem mass spectrometry analysis. The peptides were pre-separated using an HPLC system (RIGOL L-3220, Rigol Technologies Co., Ltd.). After desalting, the peptides were loaded onto an Acclaim PepMap C18 trap column (150 μm × 2 cm, 5 μm, 100 Å; Thermo Fisher Scientific) connected to a Dionex Ultimate 3,000 nano liquid chromatography system. Then, the peptides were resolved on an analytical C18 reversed-phase column (75 μm × 15 cm, 5 μm, 300 Å; Agela Technologies). Elution was achieved with a 4–90% (v/v) acetonitrile gradient in 0.1% formic acid over 105 min at a flow rate of 400 nL/min, and the eluted peptides were analyzed on a Orbitrap Fusion, mass spectrometer (Thermo Fisher Scientific, Waltham, MA, USA). Operated in positive ion and data-dependent acquisition mode. Full mass spectrometry scans were acquired at a resolution of 70,000, followed by tandem mass spectrometry scans, which were acquired at a resolution of 35,000. Fragmentation was achieved using higher-energy collisional dissociation with an isolation window of 2.0 Da and a minimum signal threshold of 1 × 10^5^. Normalized collision energy was set to 38, with a stepped collision energy of ±20% to optimize the efficiency of higher-energy collisional dissociation. For protein identification, the raw data were searched against the *S. aureus* reference proteome available at UniProt. Searches were performed using Proteome Discoverer 2.4 search engines, with the following thresholds: false discovery rate < 1% at both peptide and protein levels, minimum of one unique peptide per protein, and a precursor mass tolerance of 15 ppm. The identified proteins were further subjected to Gene Ontology (GO) enrichment analysis using the DAVID online platform and functional pathway annotation using the Kyoto Encyclopedia of Genes and Genomes (KEGG) database. The results enabled the visualization of the distribution of differentially expressed proteins (DEPs) across relevant biological pathways (Baldan-Martin et al., 2017).

### 2.8 Erythrocytolysis test

A 4% suspension of rabbit red blood cells (Solarbio, China; Cat# S9452), derived from the fresh peripheral blood of a single healthy rabbit, was used in this experiment. The suspension was diluted with sterile PBS to a final concentration of 2% and subsequently mixed with each of the three EV samples in 1.5 mL Eppendorf tubes. The mixtures were shaken gently in a thermostatic oscillator set to 37°C and 220 rpm. Optical density was measured at 620 nm (OD_620_) at 1, 2, 3, 4, 6, and 8 h post-treatment. Cell survival rate was determined from the OD_620_ value, and the hemolytic activity of the three groups of EVs on red blood cells was assessed. Cell survival rate = (experimental group OD_620_ − blank group OD_620_)/(control group OD_620_ − blank group OD_620_) (Chen et al., 2023).

### 2.9 Biofilm formation experiment

The effect of EVs on the biofilm-forming ability of OS-MRSA strains was assessed using the biofilm formation assay. Cells (approximately 2 × 10^5^ colony forming units) in the logarithmic growth phase were seeded in 96-well plates in 200 μL of TSB and incubated with EVs at 37°C for 24 h. The culture medium was discarded, and each well was refilled with 200 μL of 1% crystal violet. The plates were incubated at 37°C for 30 min, then rinsed twice with sterile water to discard the dye. The biofilms were dissolved in 200 μL of 95% ethanol, and the OD_620_ values of the supernatants (OD_planktonic_) were measured (n = 3). The TSB medium group served as the negative control group (OD_CV control_, where CV denotes crystal violet). The effect of EVs on the biofilm-forming ability of OS-MRSA was evaluated in terms of the biofilm formation index, which was calculated using the following formula: (OD_CV biofilm_ – OD_CV control_)/OD_planktonic_ (He et al., 2017).

### 2.10 Broth microdilution assay

Hundred microliter of oxacillin, serially diluted within the range of 0.032–16 μg/mL, along with MH broth (Solarbio, China), were added to the wells of a 96-well plate. The logarithmic phase suspension of OS-200 bacteria was standardized to 0.5 McFarland and inoculated into the 96-well plate, containing various concentrations of oxacillin, at a final concentration of 1.5 × 10^5 CFU/mL per well. Positive control wells, containing only MH broth and bacterial suspension, and negative control wells, containing only MH broth, were also prepared. Three experimental groups were established, namely, the EV_control_ group (with the addition of 10 μL PBS), the EV_1/8MIC_ group (with the addition of 10 μL of 1/8MIC EVs), and the EV_1/2MIC_ group (with the addition of 10 μL of 1/2MIC EVs). After 24 h of static incubation at 37°C, the minimum inhibitory concentration (MIC) was determined by visually observing the lowest concentration of oxacillin that inhibited visible bacterial growth (Saravolatz et al., 2014).

### 2.11 Cell proliferation experiment

A549 human lung adenocarcinoma epithelial cells (BNCC, China) were seeded in 96-well plates at a density of 5 × 10^3^ cells per well and cultured for 24 h in Dulbecco’s modified Eagle medium (Giboc, Suzhou, China) supplemented with 10% fetal bovine serum (VivaCell, Shanghai, China). Subsequently, the cells were treated with EVs at either one of two volumes (10 μL or 30 μL) and either one of two concentrations (10 μg/mL or 30 μg/mL). The protein content of the EVs was quantified using the Bradford assay and normalized using sterile PBS. Cells cultured in the absence of EVs and wells only containing the medium served as the negative control and blank groups, respectively. After 24 h of EVs treatment, cells were incubated for 1 h with 10 μL of Cell Counting Kit (CCK)-8 reagent (Biosharp, Beijing, China), and the absorbance was measured at 450 nm using a microplate reader. Cell viability (%) was calculated using the following formula: Cell viability (%) = (OD_450_ of treatment group – OD_450_ of blank group)/(OD_450_ of NC group – OD_450_ of blank group) × 100% (Li et al., 2023), where NC denotes negative control.

### 2.12 Cell tracer experiment

This qualitative study investigated the cellular uptake potential of EVs. EVs were fluorescently labeled with PKH67 (Sigma, Germany) using the following protocol. Briefly, EVs were resuspended in Diluent C provided in the PKH67 labeling kit, mixed with the dye solution, and incubated at room temperature for 5 min. The staining reaction was terminated by adding 4 mL of 1% bovine serum albumin (YuanYe Bio, China). The labeled EVs were washed with PBS and ultracentrifuged at 100,000 × *g* for 1 h to remove the unbound dye, yielding three labeled EV preparations: PKH67-EV_control_, PKH67-EV_1/2MIC_, and PKH67-EV_1/8MIC_. A549 cells cultured on coverslips in 24-well plates were incubated separately with 10 μL of each of the PKH67-labeled EVs. The culture medium was removed after 24 h of incubation. The cells were washed with PBS, fixed in 4% paraformaldehyde (Solarbio, China) at room temperature for 20 min, and permeabilized with 0.1% Triton X-100 (Sigma, Germany) at room temperature for 20 min. The actin cytoskeleton was stained using SF594-conjugated phalloidin (Solarbio, China), and the nuclei were counterstained with an antifade mounting medium containing 4′,6-diamidino-2-phenylindole (Solarbio, China). Fluorescence images were acquired using a laser scanning confocal microscope (Gao et al., 2024).

### 2.13 Detection of apoptosis by flow cytometry

The effects of EVs on cell apoptosis were investigated using flow cytometry. Adherent A549 cells were incubated separately with the three EV preparations for 24 h, with two volumes tested for each EV preparation (10 μL and 30 μL). Subsequently, the cells were stained with fluorescein isothiocyanate–conjugated annexin V and propidium iodide (BD Biosciences). Flow cytometric analysis was performed immediately using the CellQuest Pro software (BD Biosciences) (Ye et al., 2025).

### 2.14 Detection of inflammatory factors

Log-phase RAW 264.7 cells (BNCC, China) were seeded in 6-well plates at a density of 1 × 10^6^ cells per well, incubated for 12 h, and stimulated for 12 h with 100 μg/mL of phorbol-12-myristate-13-acetate (Solarbio, China) to induce differentiation into a macrophage-like phenotype. During this time window, more than 80% of the cells were confirmed to be adherent through microscopic observation. Following differentiation, the cells were divided into three groups: a control group treated with an equivalent volume of PBS (without EVs) and two experimental groups treated with EVs at concentrations of 5 μg/mL and 20 μg/mL. After 6 h of treatment, the culture supernatants from each group were collected, and the levels of tumor necrosis factor-*α* (TNF-α) and interleukin-6 (IL-6) were measured using enzyme-linked immunosorbent assay kits (Shanghai Enzyme-linked Biotechnology Co., Ltd., China) (Li et al., 2023).

### 2.15 Statistical analysis

Data were analyzed using the SPSS 26.0 software. Statistical analyses were performed using Student’s t test, Mann–Whitney test, or two-way analysis of variance (Tukey’s multiple-comparison test). *p* < 0.05 was considered to denote a significant difference. Statistical charts were plotted using GraphPad Prism 10.0 software.

## 3 Results

### 3.1 Sub-inhibitory concentrations of oxacillin stimulated the secretion of OS-MRSA EVs

**Figure 1:**
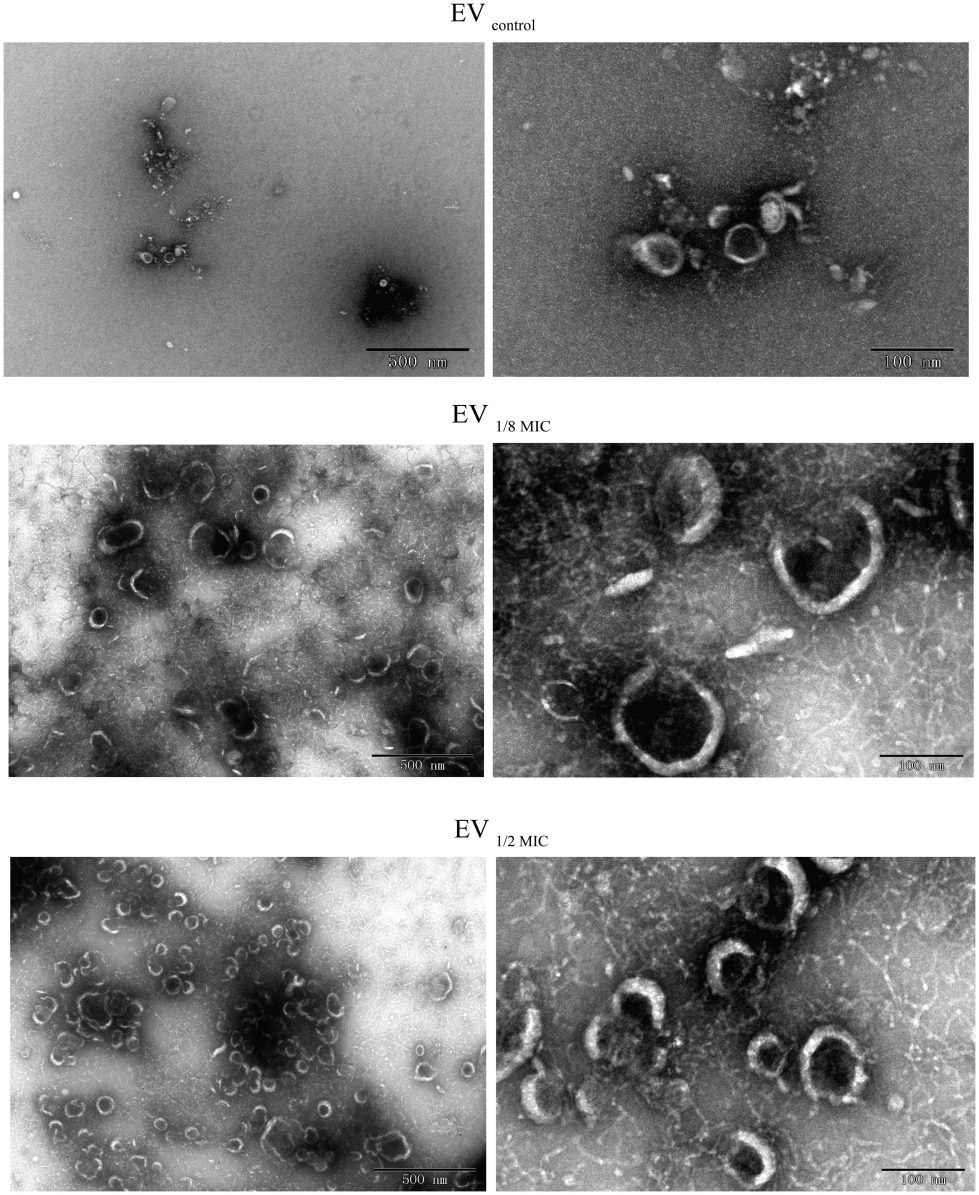
TEM revealing disk-shaped EVs secreted by OS-200 strain. TEM images of EVs. Scale bars: 500 nm **(left)**, 100 nm **(right)**.

**Figure 2:**
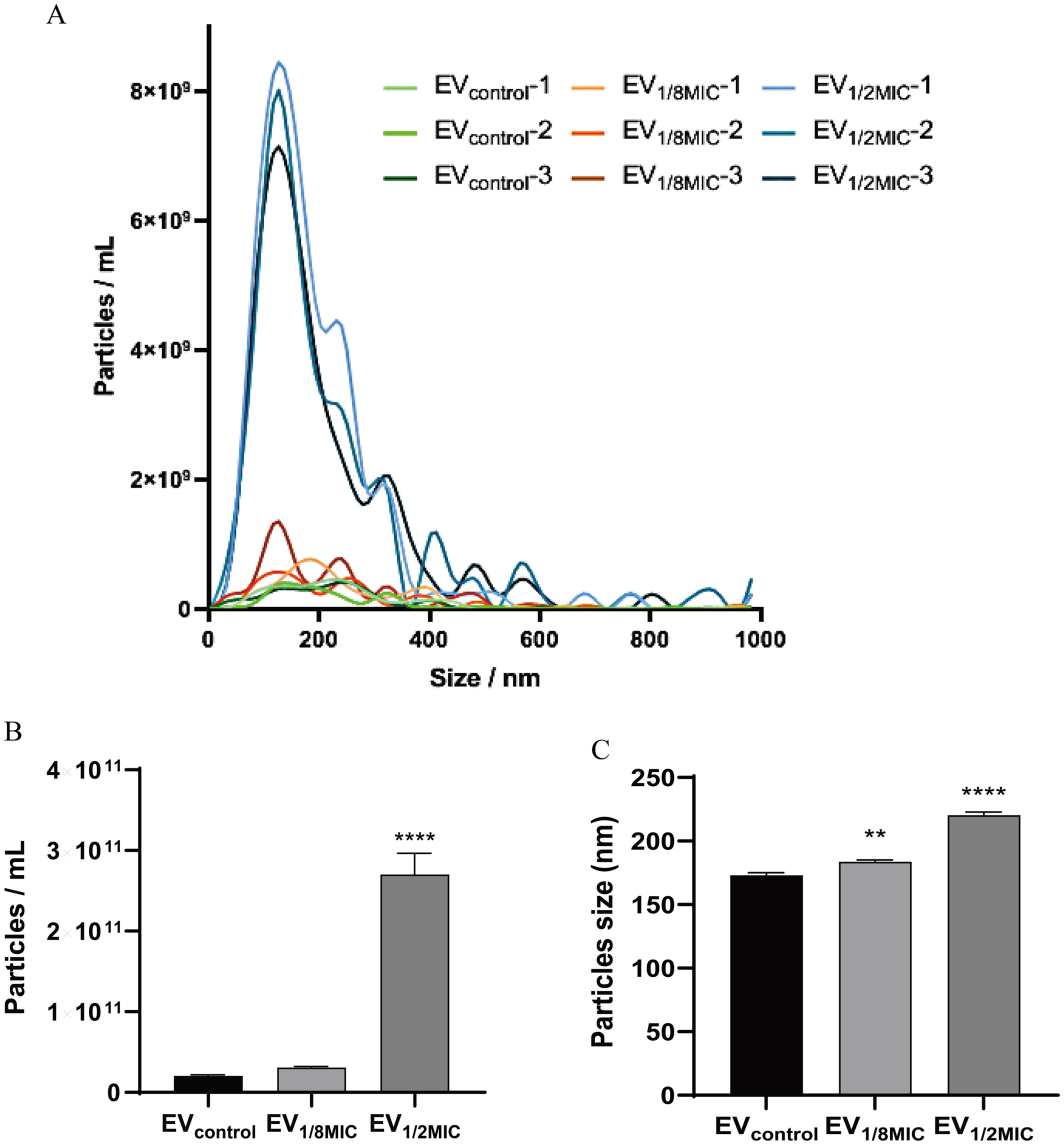
NTA of EVs secreted by *S. aureus* strain OS-200. **(A)** NTA measurements of EVs (*n* = 3). **(B)** Mean particle count of EVs (*n* = 3). **(C)** Mean particle size of EVs (*n* = 3). Data: mean ± SEM; Student’s *t*-test: **p < 0.01, ***p < 0.001, ****p < 0.0001.

**Figure 3:**
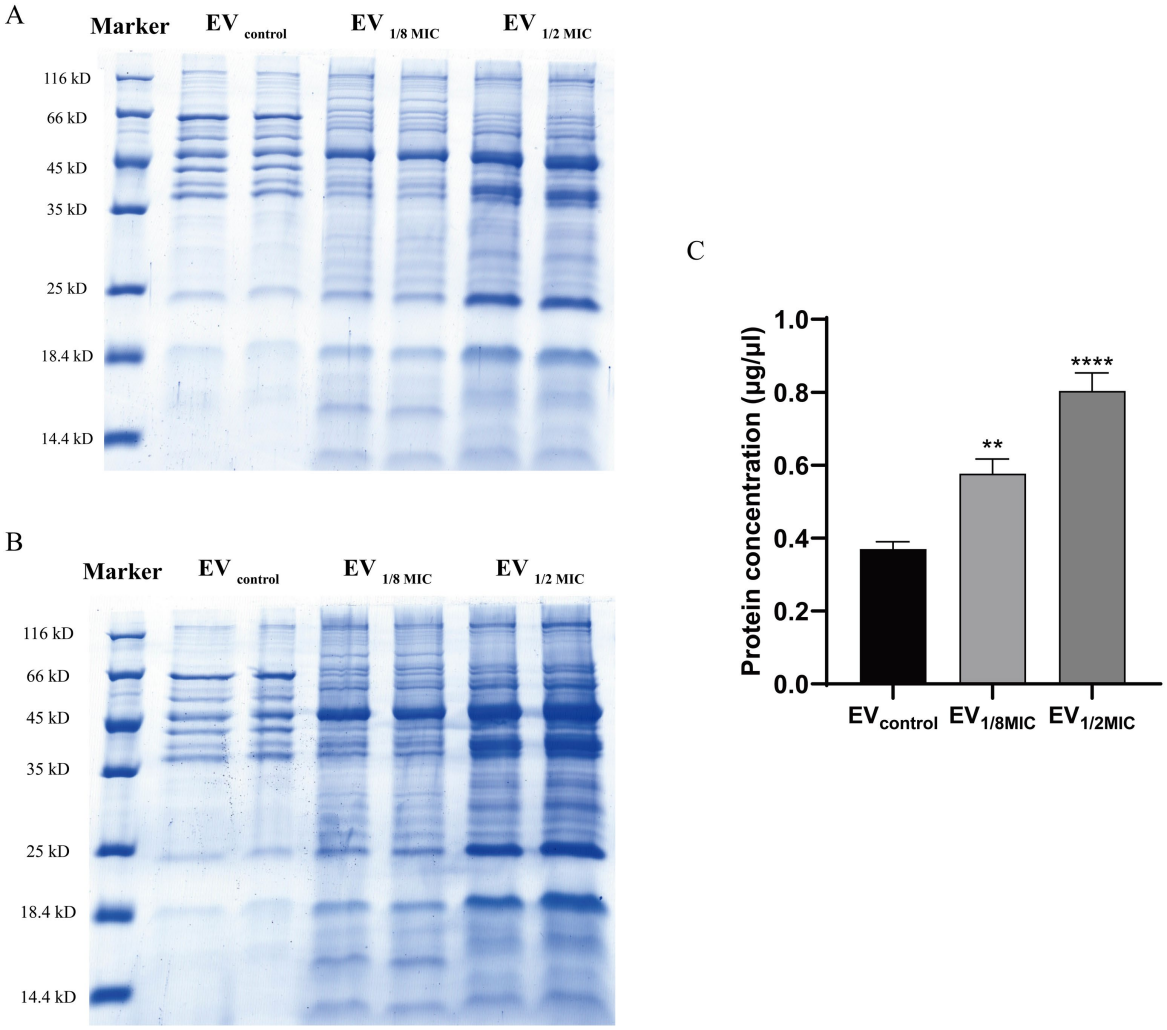
Protein yield and Coomassie-stained protein profile of OS-200 EVs. **(A,B)** SDS-PAGE with Coomassie Brilliant Blue staining of EVs. Three EV preparations under both loading conditions: **(A)** equal protein mass (10 μg) and **(B)** equal volume (20 μl). **(C)** Quantification of EVs protein content using Bradford assay. Data: mean ± SEM; Student’s *t*-test: **p < 0.01, ***p < 0.001, ****p < 0.0001. Lanes were spliced to remove empty spaces and align labels.

The EVs used in this study were isolated from culture supernatants of the OS-200 strain. Transmission electron microscopy revealed that EVs from all three experimental groups—EV_1/8MIC_, EV_1/2MIC_, and EV_control_—exhibited similar disc-like morphologies, irrespective of oxacillin exposure (Figure 1). However, nanoparticle tracking analysis demonstrated that OI-EVs had significantly higher particle counts and larger average sizes than EV_control_. The particle count and average particle size of EV_control_ were (2.03 ± 0.15) × 10^10^ particles/ml and 173.1 ± 89 nm, respectively (calculated from 2,504 particles); for EV_1/8MIC_, the corresponding metrics were (3.07 ± 0.15) × 10^10^ particles/ml and 183.7 ± 152.5 nm, respectively (calculated from 1,547 particles); and for EV_1/2MIC_, the corresponding metrics were (27 ± 2.65) × 10^10^ particles/ml and 220.4 ± 194.8 nm, respectively (calculated from 1, 289 particles). The trend of particle counts and particle size followed the order EV_control_ < EV_1/8MIC_ < EV_1/2MIC_; *p* < 0.05 (Figure 2). Furthermore, sodium dodecyl sulfate–polyacrylamide gel electrophoresis analysis revealed distinct protein profiles for all three EVs within the molecular weight range of 18–66 kDa under both equal-protein loading (10 μg each; Figure 3A) and equal-volume loading (20 μL each; Figure 3B). Notably, the protein content of OI-EVs increased with oxacillin concentration (Figure 3C), a phenomenon that can be attributed to the prominent increase in particle count and size observed in the EV_1/2MIC_ group.

### 3.2 Proteomic comparison of EV_control_, EV_1/8MIC_, and EV_1/2MIC_

Proteomic analysis was performed to investigate the impact of oxacillin exposure on the protein composition of OI-EVs. A total of 586 proteins were identified across the three groups of tandem mass tag-labeled samples. The results demonstrated that exposure to sub-inhibitory concentrations of oxacillin significantly increased the expression of multiple proteins in OI-EVs, with the effect being more pronounced in the case of EV_1/2MIC_ (Supplementary File 2). The upregulated proteins were classified into five categories on the basis of their functions: (i) virulence proteins, such as Hlg, HlgB, CtaB, QoxB, PmtC, *β*-channel-forming cytolysin, lipoprotein cluster (Lpl9, SFAG_00213, GmpC), and LCP protein family (LcpA, gene = BSZ10_08965; LcpA, gene = QU38_05525); (ii) adhesion-related proteins, such as sortase, enolase, and DegP/HtrA; (iii) drug resistance-related proteins, such as PBP2, BlaZ, EmrB, FemA, TcaR, and HsdM; (iv) secretion-related proteins, such as SecA and SecDF; and (v) virulence regulation-related proteins, such as Agr and MgrA (Table 1). Notably, certain virulence-associated proteins were more abundant in EV_1/2MIC_ than in EV_1/8MIC_, including Hlg, HlgB, β-channel-forming cytolysin, CymR, FemA, and MgrA. These findings suggest that in OS-MRSA exposed to sub-inhibitory concentrations of oxacillin, a dose of half the MIC enhances the expression of specific virulence factors, whereas a dose of one-eighth the MIC reduces the expression of these virulence factors (Table 1).

**Table 1:** The DEPs of EV from three comparison groups (EV _control_, EV_1/8MIC_ and EV_1/2MIC_).

Protein	Gene	EV_1/2MIC_ *vs.* EV _control_		EV_1/8MIC_ *vs.* EV _control_		EV_1/2MIC_ *vs.* EV_1/8MIC_	
		Ratio	*p*_value	ratio	*p*_value	ratio	*p*_value
Delta-hemolysin	*hld*	0.288	<0.001	0.4055	<0.001	0.710	<0.05
Hemolysin gamma	*hlg*	1.4215	<0.001	–	–	2.770	<0.001
Bi-component gamma-hemolysin HlgAB/HlgCB subunit B	*hlgB*	1.3935	<0.001	0.7365	<0.001	1.892	<0.001
Protoheme IX farnesyltransferase	*ctaB*	2.2545	<0.001	1.706	<0.001	-	-
Quinol oxidase subunit 1	*qoxB*	2.0605	<0.001	1.473	<0.001	1.399	<0.001
Phenol-soluble modulin PSM-alpha	*psm α*	0.7255	<0.001	0.4825	<0.001	1.504	<0.001
Cysteine metabolism repressor	*cymR*	1.1415	<0.001	0.473	<0.001	2.413	<0.001
Phenol-soluble modulin export ABC transporter ATP-binding protein PmtC	*pmtC*	5.3315	<0.001	2.083	<0.001	2.560	<0.001
Beta-channel forming cytolysin (leucocidin homolog)	G0Z62_12290	1.731	<0.001	0.622	<0.001	2.783	<0.001
Alpha/beta hydrolase	E3A28_05360	2.6945	<0.001	2.183	<0.001	1.234	<0.05
	E4U00_12685	3.607	<0.001	2.5585	<0.001	1.410	<0.001
Lipoprotein	SAXG_01787	1.632	<0.05	1.3575	<0.05	1.202	<0.001
Membrane lipoprotein	*lpl9*	1.5665	<0.05	1.5335	<0.001	–	–
Lipoprotein	*gmpC*	1.812	<0.05	–	–	–	–
Putative lipoprotein	SFAG_00213	2.1595	<0.05	2.4115	<0.05	0.896	<0.001
Enolase	*eno*	2.0255	<0.001	–	–	7.676	<0.001
Class A sortase SrtA	*srtA*	2.127	<0.05	2.3695	<0.001	0.898	<0.05
Serine protease, DegP/HtrA, do-like protein	*degP*	2.142	<0.001	1.9255	<0.05	–	–
LytR_cpsA_psr domain-containing protein (LCP family protein)	BSZ10_08965	2.691	<0.05	1.751	<0.05	–	–
	QU38_05525	3.9075	<0.05	–	–	31.192	<0.05
	HUW54_06505	1.346	<0.001	0.9665	<0.001	1.393	<0.05
Penicillin-binding protein 2	KMZ21_07315	2.939	<0.05	1.8015	<0.05	1.631	<0.001
Beta-lactamase	*blaZ*	2.2845	<0.001	3.4135	<0.001	0.669	–
	SHAG_01009	1.6435	<0.001	–	–	3.376	<0.001
FemA	*femA*	1.477	<0.001	0.9515	<0.001	1.552	<0.001
Drug resistance transporter	*emrB*	2.737	<0.001	2.599	<0.001	–	–
Teicoplanin-resistance associated HTH-type transcriptional regulator TcaR	*tcaR*	1.5725	<0.05	0.642	<0.001	2.449	<0.001
Site-specific DNA-methyltransferase (adenine-specific)	*hsdM*	2.653	<0.001	–	–	26.711	<0.001
Type VII secretion system accessory factor EsaA	*esaA*	0.3205	<0.05	0.6915	<0.001	0.463	<0.05
Multifunctional fusion protein	*secDF*	2.7245	<0.001	1.6805	<0.001	1.621	<0.001
Preprotein translocase subunit SecA	*secA*	1.8705	<0.05	–	–	4.643	<0.001
HTH-type transcriptional regulator MgrA	*mgrA*	1.353	<0.05	0.5765	<0.001	2.347	<0.001
Transcriptional regulator MraZ	*mraZ*	0.693	<0.001	0.328	<0.001	2.113	<0.001
Accessory gene regulator A	*agrA*	1.975	<0.05	0.6225	<0.001	3.173	<0.001
Chaperonin GroEL	*groL*	0.4155	<0.05	1.336	<0.001	0.311	<0.001
Probable transglycosylase IsaA	*isaA*	0.2625	<0.05	0.353	<0.05	–	–
N-acetylmuramoyl-L-alanine amidase	*amiD*	0.484	<0.05	0.436	<0.001	–	–

Protein with multiple difference of 1.2 or 0.8 (*p* < 0.05); non-significant difference (*p* > 0.05) without specific values of ratio and p, indicated by the symbol “–”.

#### 3.2.1 EV_1/2MIC_ *vs.* EV_control_

**Figure 4:**
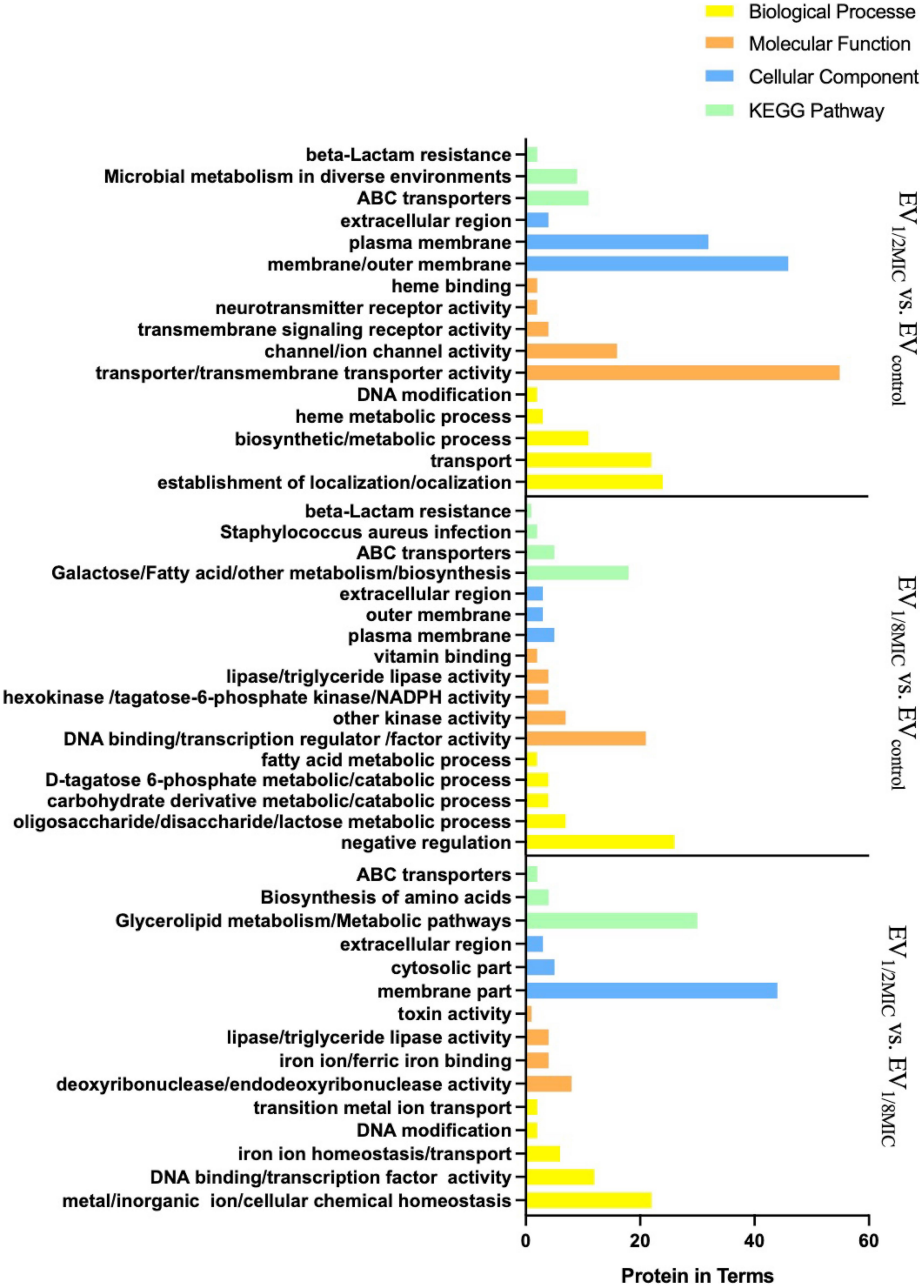
GO and KEGG (key pathways) enrichment profiling of DEPs from the three experimental groups (EV_1/2MIC_ *vs.* EV_control_, EV_1/8MIC_ *vs.* EV_control_, and EV_1/2MIC_ *vs.* EV_1/8MIC_). (i) EV_1/2MIC_ *vs.* EV_control_. BP: related to protein localization establishment, macromolecule transport, DNA modification, amino acid metabolism (including phenylalanine and taurine metabolism), and heme metabolic processes, MF: key enriched activities encompassed activity of transmembrane transporters (such as ABC transporters), ion channel gating, transmembrane signaling receptor activity, neurotransmitter receptor binding, phosphofructokinase activity, heme binding, and protoheme IX farnesyltransferase activity. CC: localization to the cell membrane, plasma membrane, extracellular region, and respiratory chain complexes. KEGG: ABC transporters, microbial metabolism in diverse environments, and β-lactam resistance. (ii) EV_1/8MIC_ *vs.* EV_control_. BP: carbohydrate metabolic processes (such as glycolysis and galactose metabolism), fatty acid metabolism, negative regulation of RNA biosynthesis, and transcription. MF: lipase activity, hexokinase activity, NADPH dehydrogenase activity, DNA-binding transcription factor activity, and fructokinase activity. CC: localization to the outer membrane, plasma membrane, and extracellular space. KEGG: ABC transporters, *S. aureus* infection with downregulated cytolysin, and β-lactam resistance. (iii) EV_1/2MIC_ *vs.* EV_1/8MIC_. BP: cellular ion homeostasis including iron and cations, chemical homeostasis, and toxin-mediated processes, such as hemolysis and cytolysis. MF: ferric iron binding, toxin activity associated with β-channel-forming cytolysin, DNA-binding transcription factor activity, and hydrolase/transferase activities. CC: localization to the outer membrane and extracellular region. KEGG: metabolism, biosynthesis of amino acid, and ABC transporters.

The results of GO enrichment analysis (Figure 4) indicated that DEPs were significantly enriched in (i) biological processes (BPs) related to protein localization establishment, macromolecule transport, DNA modification, biosynthesis/metabolism, and heme metabolic processes; (ii) molecular functions (MFs) related to activity of transmembrane transporters such as ATP-binding cassette (ABC) transporters, ion channel activity, transmembrane signaling receptor activity, neurotransmitter receptor activity, heme binding, and protoheme IX farnesyltransferase activity; (iii) cellular components (CC) analysis indicated predominant localization of DEPs to outer membrane, plasma membrane, and extracellular region. Notably, CtaB (heme IX farnesyltransferase) was upregulated and functionally associated with heme metabolism (BP), heme IX farnesyltransferase activity (MF), and plasma membrane localization (CC). Similarly, QoxB (quinol oxidase subunit) was upregulated and associated with aerobic respiration (BP), heme binding (MF), and localization to the cell membrane/respiratory chain (CC).

**Figure 5:**
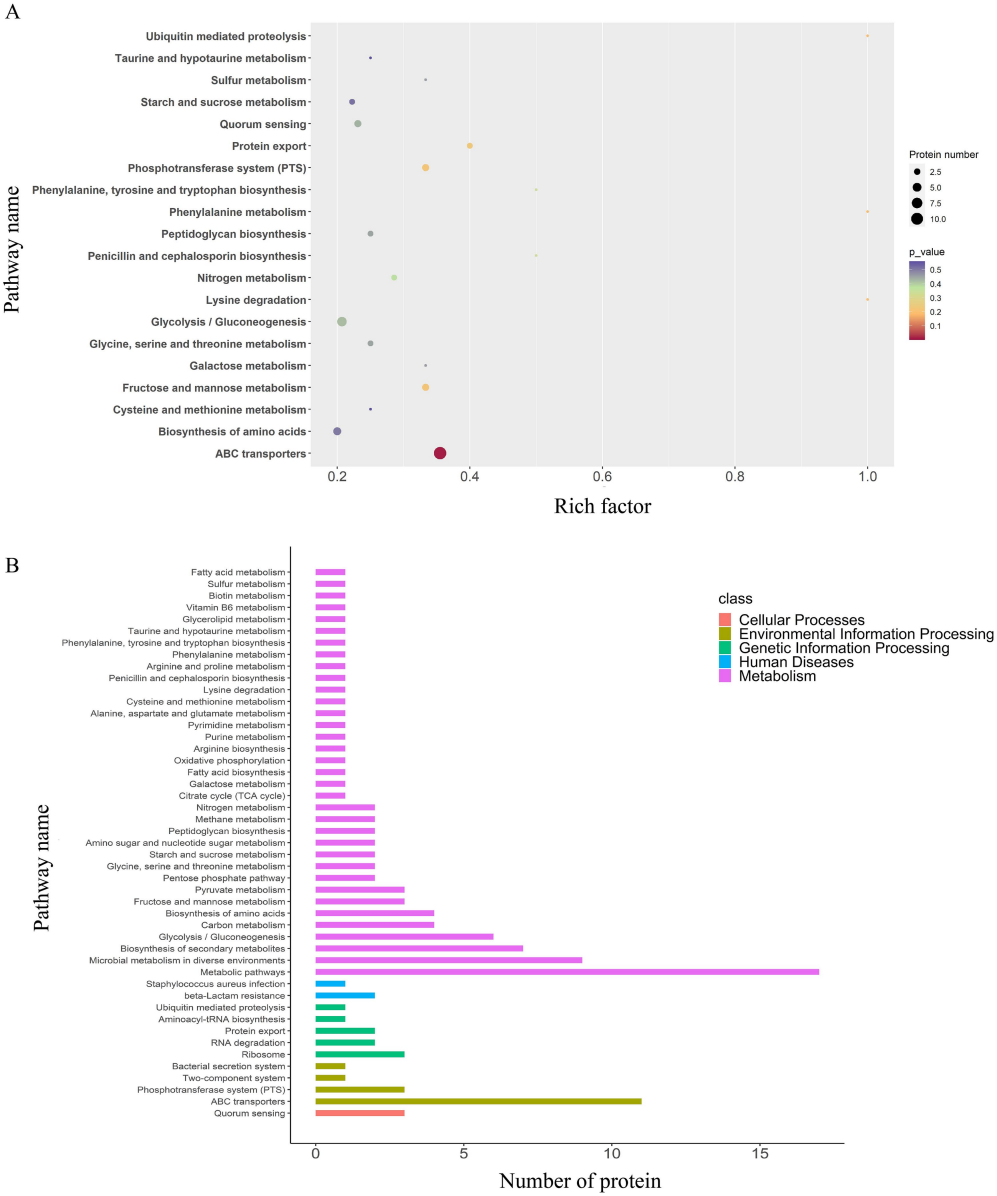
KEGG pathway enrichment and classification analysis of DEPs in the EV_1/2MIC_ *vs.* EV_control_ groups. **(A)** Top 20 DEPs identified in 48 enriched pathways, predominantly involving ABC transporters, microbial metabolism in diverse environments, quorum sensing, and β-lactam resistance where key resistance proteins PBP2 and BlaZ were both upregulated. Pathway categorization revealed **(B)** KEGG pathway classification analysis: metabolism pathways (75%), including microbial metabolic adaptation (such as carbon metabolism and glycolysis/gluconeogenesis); human diseases (4%), including β-lactam resistance with upregulated PBP2 and BlaZ along with *S. aureus* infection featuring upregulated cytolysin; genetic information processing (10%), involving ribosome, RNA degradation, and protein export; environmental information processing (8%), involving ABC transporters and bacterial secretion system; and cellular processes (2%), primarily involving quorum sensing.

A total of 48 enriched pathways were identified using KEGG pathway analysis (Figures 4, 5), predominantly involving ABC transporter signaling, microbial metabolism in diverse environments, quorum sensing, and *β*-lactam resistance. The two key resistance proteins—penicillin-binding protein 2 (PBP2) and *β*-lactamase (BlaZ)—were both upregulated. The enriched pathways were categorized as follows: (i) metabolism pathways (75%), including microbial metabolic adaptation such as carbon metabolism and glycolysis/gluconeogenesis; (ii) human diseases (4%), including *β*-lactam resistance with upregulated PBP2 and BlaZ and *S. aureus* infection with upregulated cytolysin; (iii) genetic information processing (10%), involving ribosomal pathways, RNA degradation, and protein export; (iv) environmental information processing (8%), involving ABC transporters and the bacterial secretion system; and (v) cellular processes (2%), primarily involving quorum sensing.

#### 3.2.2 EV_1/8MIC_ *vs.* EV_control_

Gene Ontology enrichment analysis (Figure 4) revealed that in the BP domain, the differentially expressed proteins were enriched in carbohydrate metabolic processes such as glycolysis and galactose metabolism, fatty acid metabolism, and negative regulation; in the MF domain, they were enriched in lipase activity, hexokinase activity, nicotinamide adenine dinucleotide phosphate activity, DNA binding, transcription regulation, and factor activity; in the CC domain, they were found to be predominantly localized to the outer membrane, plasma membrane, and extracellular region.

**Figure 6:**
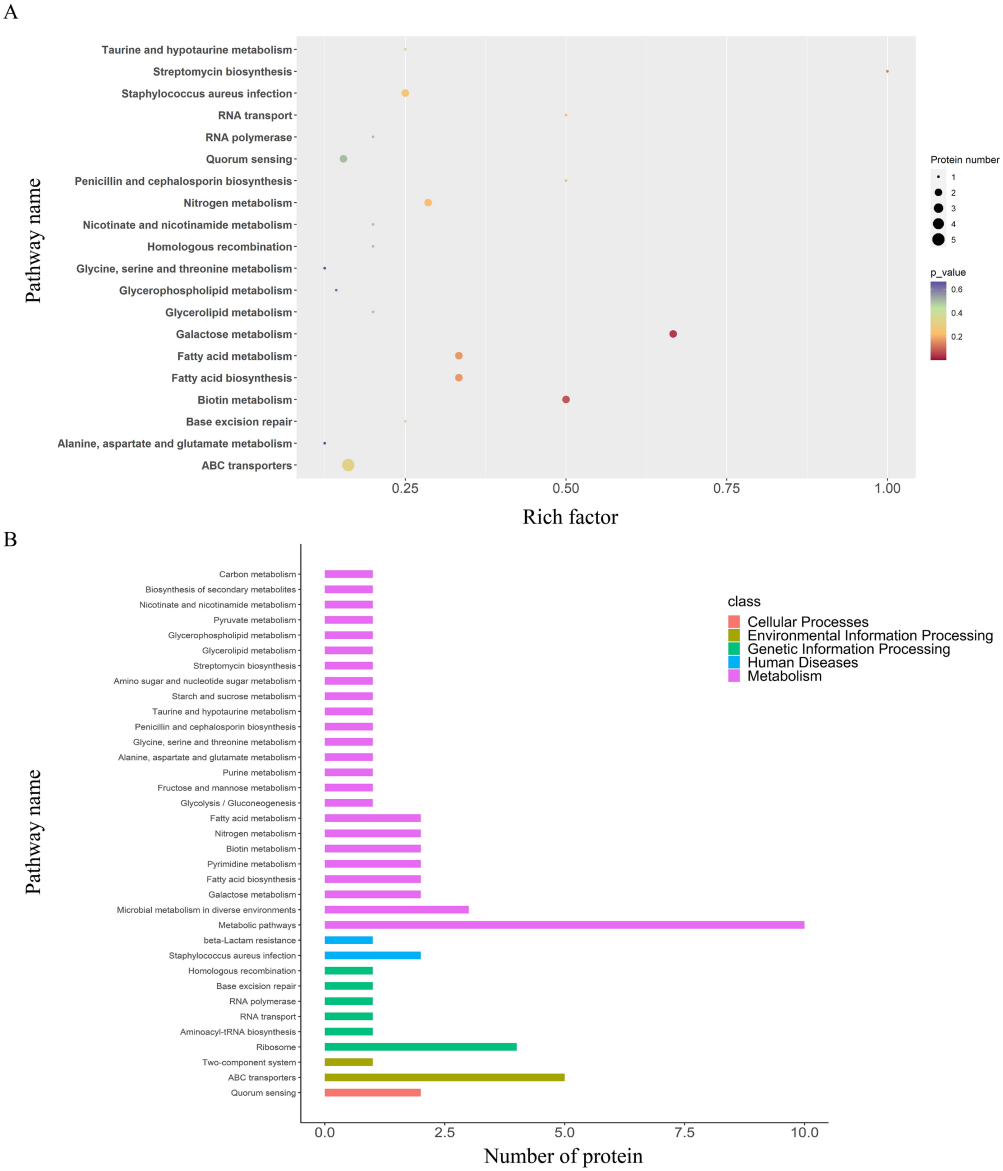
KEGG pathway enrichment and classification analysis of DEPs in the EV_1/8MIC_ *vs.* EV_control_ groups. **(A)** Top 20 DEPs in 36 enriched pathways, including ABC transporters, *S. aureus* infection with downregulated cytolysin, and β-lactam resistance where only BlaZ was upregulated. **(B)** KEGG pathway classification analysis: metabolism (69%), involving microbial metabolic adaptation and fatty acid biosynthesis; human diseases (6%), including β-lactam resistance with upregulated BlaZ and *S. aureus* infection with downregulated cytolysin; genetic information processing (17%), including RNA transport and base excision repair; environmental information processing (6%), including ABC transporters and two-component system; and cellular processes (3%), involving quorum sensing.

A total of 36 enriched pathways were identified using KEGG pathway analysis (Figures 4, 6), involving ABC transporters, *S. aureus* infection with downregulated cytolysin, and *β*-lactam resistance where only BlaZ was upregulated. The enriched pathways were categorized as follows: (i) metabolism pathways (69%), involving microbial metabolic adaptation and fatty acid biosynthesis; (ii) human diseases (6%), including β-lactam resistance with upregulated BlaZ and *S. aureus* infection with downregulated cytolysin; (iii) genetic information processing (17%), including RNA transport and base excision repair; (iv) environmental information processing (6%), including ABC transporters and two-component systems; and (v) cellular processes (3%), mainly involving quorum sensing.

#### 3.2.3 EV_1/2MIC_ *vs.* EV_1/8MIC_

Gene Ontology enrichment analysis (Figure 4) indicated that in the BP domain, the differentially expressed proteins were enriched in cellular chemical homeostasis, iron ion homeostasis/transport, and DNA modification; in the MF domain, they were enriched in ferric iron binding, toxin activity associated with β-channel–forming cytolysin, and DNA-binding transcription factor activity; in the CC domain, they were found to be predominantly localized to the membrane, cytosolic part, and extracellular region. β-channel–forming cytolysin, a key virulence protein, was found to be upregulated with functional associations with toxin activity (MF), hemolysis (BP), and extracellular region localization (CC). Among the three EVs, the expression levels of this protein followed the order EV_1/2MIC_ > EV_control_ > EV_1/8MIC_.

**Figure 7:**
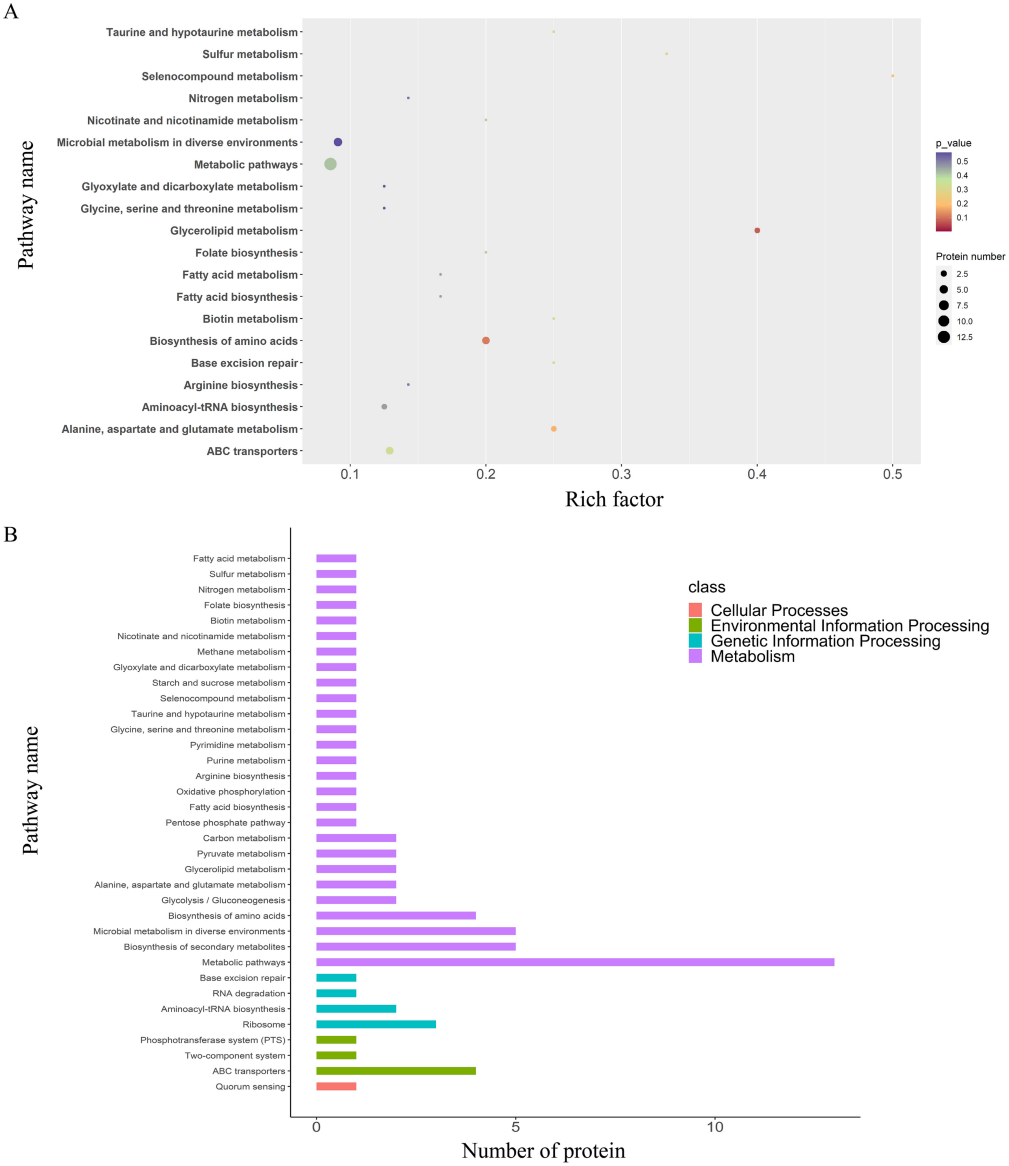
KEGG pathway enrichment and classification analysis of DEPs in the EV_1/2MIC_ *vs.* EV_1/8MIC_ groups. **(A)** Top20 of pathway enrichment of DEPs: 35 enriched pathways primarily involving ABC transporters, quorum sensing, and amino acid metabolism. **(B)** KEGG pathway classification analysis: metabolism (77%), involving amino acid and fatty acid biosynthesis and degradation; genetic information processing (11%), including RNA degradation and base excision repair; environmental information processing (9%), including ABC transporters and phosphotransferase system; and cellular processes (3%), involving quorum sensing.

A total of 35 enriched pathways were identified using KEGG pathway analysis (Figures 4, 7), primarily involving metabolism, biosynthesis of amino acids, and ABC transporters. The enriched pathways were categorized as follows: (i) metabolism (77%), involving amino acid and fatty acid biosynthesis and degradation; (ii) genetic information processing (11%), involving RNA degradation and base excision repair; (iii) environmental information processing (9%), including ABC transporters and the phosphotransferase system; and (iv) cellular processes (3%), predominantly involving quorum sensing.

### 3.3 OI-EVs exhibited strong hemolytic activity

**Figure 8:**
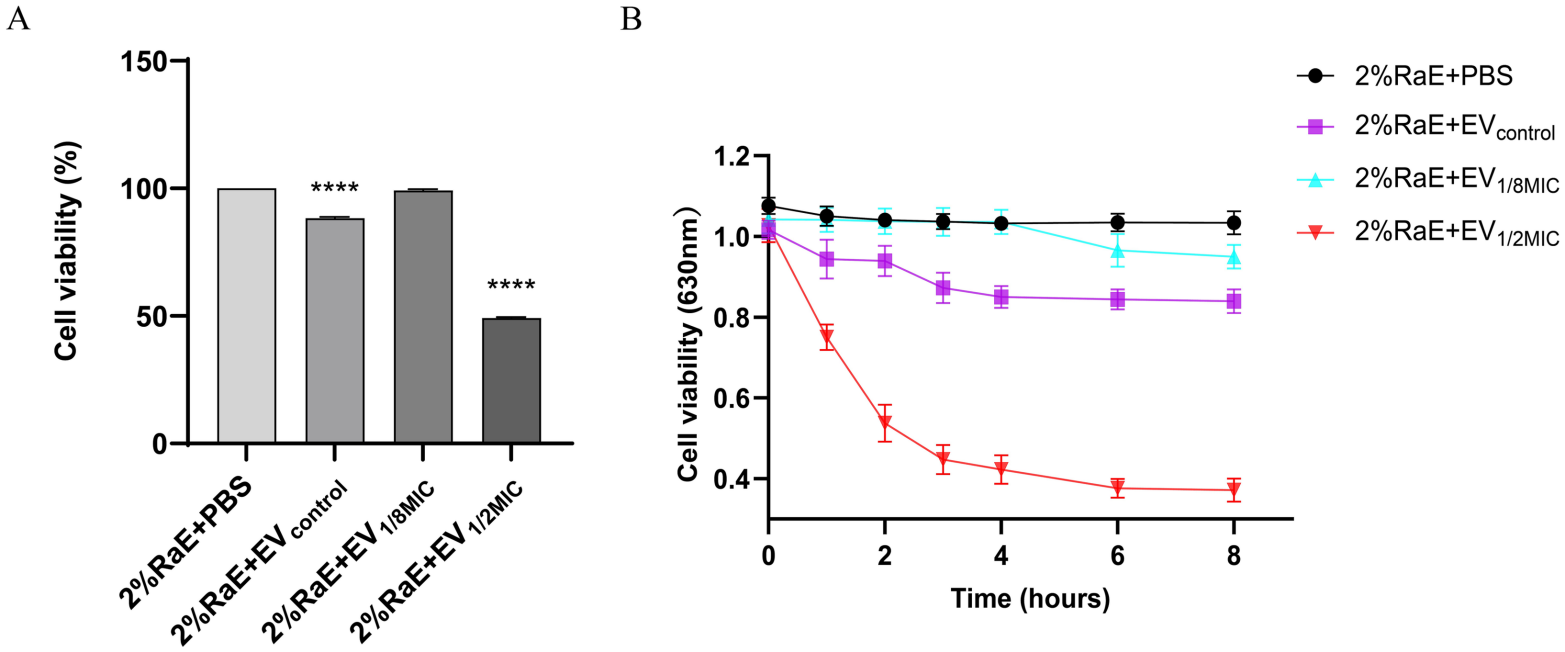
Prohemolytic effect of OI-EVs on 2% rabbit erythrocytes *in vitro*. **(A)** Cell viability at the 2-h time point. **(B)** Cell viability at the following time points: 0, 1, 2, 3, 4, 6, and 8 h. Data are presented as mean ± SEM and analyzed using Student’s *t-*test. ****p < 0.0001, ***p < 0.001, **p < 0.01, *p < 0.05.

To investigate the hemolytic activity of OI-EVs, the cell viability of rabbit erythrocytes (2%) was assessed after incubating them with the different EV preparations. Compared with the negative control group (PBS treatment), cell viability significantly decreased over time in the EV_control_ and EV_1/2MIC_ groups (*p* < 0.05). Notably, at the 2-h time point, the cell viability of the EV_1/2MIC_ group decreased by as much as 50.76%. After 2 h, the cell viability of all groups plateaued rapidly. Surprisingly, the cell viability trend in the EV_1/8MIC_ group closely mirrored that in the negative control group, showing little to no change (Figure 8).

### 3.4 OI-EVs modulated OS-MRSA physiology

#### 3.4.1 OI-EVs enhanced biofilm-forming ability of OS-MRSA

**Figure 9:**
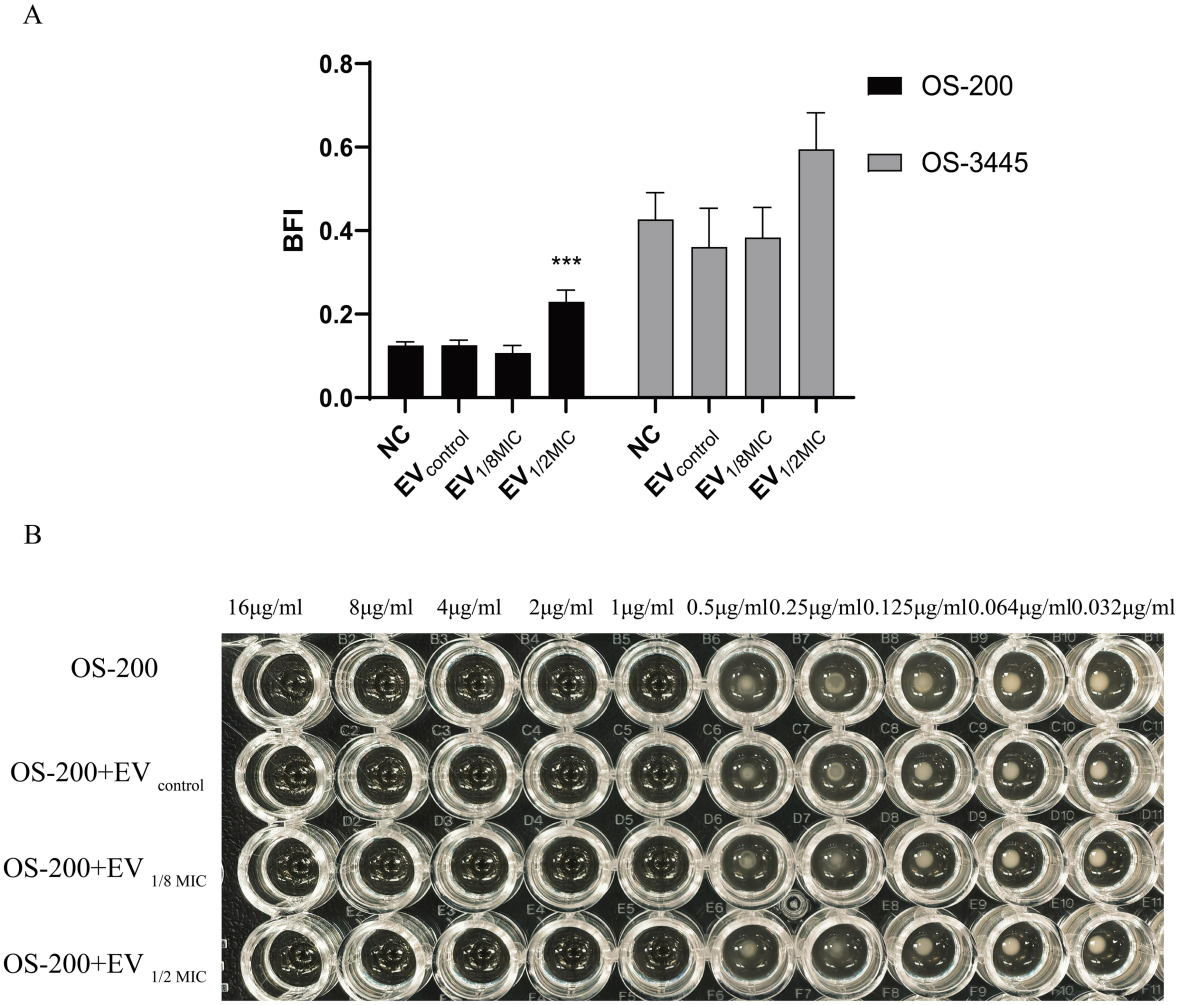
Dichotomous effects of OI-EVs on *S. aureus* phenotypes. **(A)** Biofilm matrix augmentation by EVs. **(B)** Neutral impact on oxacillin. Data are presented as mean ± SEM and analyzed using Student’s *t-*test. ***p < 0.001, **p < 0.01, *p < 0.05.

To investigate the effect of OI-EVs on bacterial biofilm formation, two OS-MRSA strains—OS-3445 (a strong biofilm-forming strain) and OS-200 (a weak biofilm-forming strain)—were incubated with the different EV preparations. Compared with the negative control group, incubation with EV_1/2MIC_ enhanced the biofilm-forming abilities of OS-200 and OS-3445 by 184% (*p* < 0.05) and 139% (*p* > 0.05), respectively. By contrast, the biofilm-forming ability of both strains remained unchanged after treatment with EV_control_ or EV_1/8MIC_ (Figure 9A).

#### 3.4.2 OI-EVs did not alter oxacillin resistance of OS-200

The effect of EVs on the oxacillin susceptibility of the OS-200 strain was assessed using the broth microdilution method. The results demonstrated that both the control group, which only contained the OS-200 strain, and the experimental groups, where the three types of vesicles were co-incubated with the strain, all exhibited identical MIC values of 1 μg/mL for oxacillin. This indicates that neither the EVs derived from the OS-200 strain nor the OI-EVs had a significant impact on the antibiotic resistance of the strain itself (Figure 9B).

### 3.5 OI-EVs altered the activities of A549 lung epithelial cells

#### 3.5.1 OI-EVs inhibited the proliferation of A549 cells

**Figure 10:**
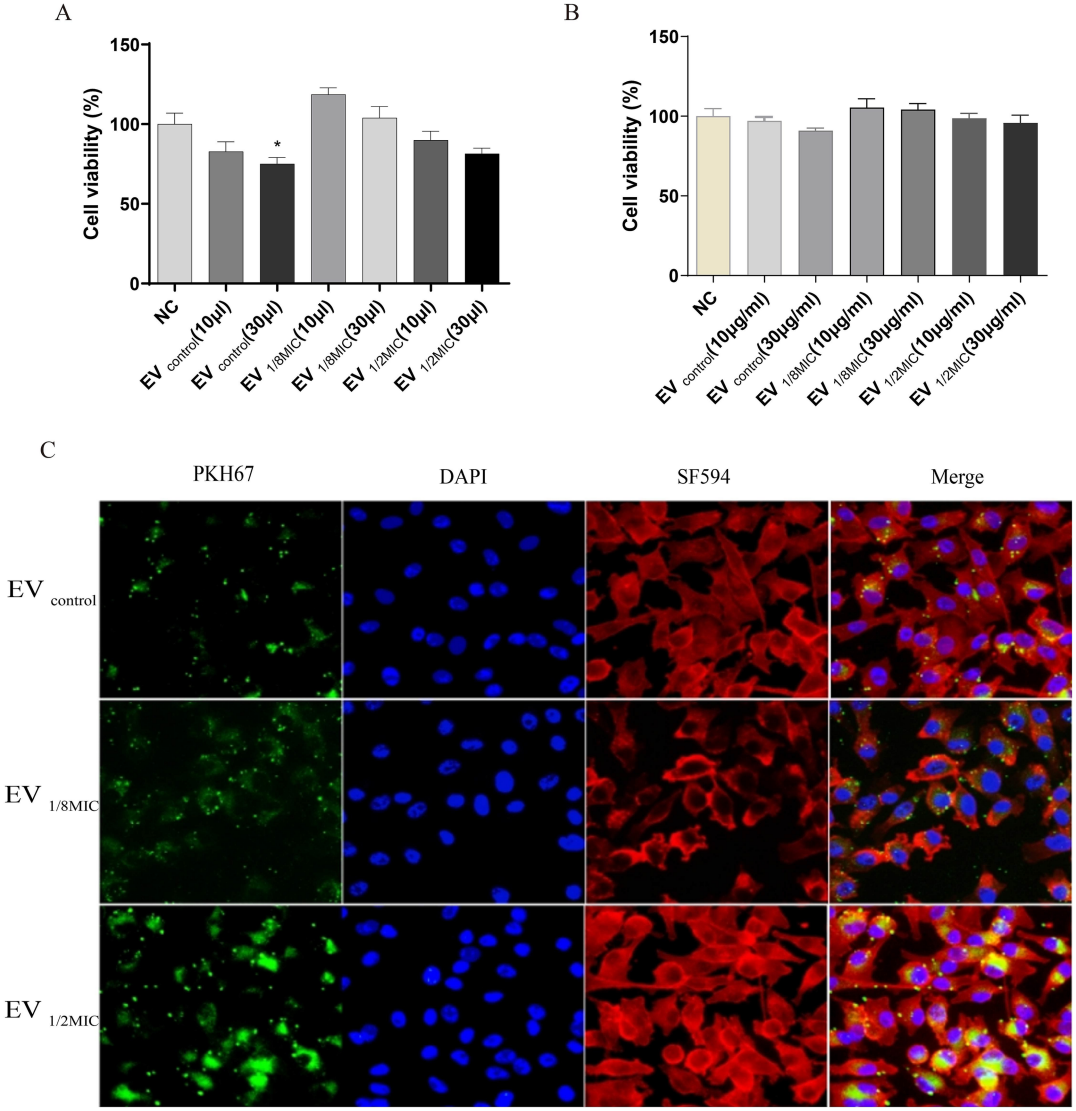
Dual effects of OI-EVs on A549 host cells: proliferation potentiation and cellular invasion. **(A)** Enhanced proliferation of EV-treated A549 cells (24 h). **(B)** Internalization of PKH67-labeled EVs into the cytoplasm of A549 cells. (Confocal microscopy: EVs, green; cytoskeleton, red; nuclei, blue).

Building on the proteomics findings, we further investigated the functional changes induced by OI-EVs. OI-EVs exerted volume-dependent cytotoxicity, as evidenced by the results of the CCK-8 assay. We examined the cytotoxic effects of two volumes (10 μL and 30 μL) and two concentrations (10 μg/mL and 30 μg/mL) of EVs. The two salient findings from this experiment were as follows. (1) Treatment with a higher volume of EVs (30 μL) significantly suppressed A549 proliferation (Figure 10A). The viabilities of cells treated with EV_control_ and EV_1/2MIC_ reduced to 75.04% (*p* < 0.05) and 81.47% (*p* > 0.05), respectively, whereas treatment with EV_1/8MIC_ did not reduce cell viability. (2) Treatment with different concentrations of EVs did not alter the proliferative outcomes (Figure 10B).

#### 3.5.2 OI-EVs possessed stronger cell penetration capabilities

Immunofluorescence staining visually demonstrated the enhanced cellular invasive capabilities of OI-EVs compared with EV_control_. OI-EVs exhibited markedly increased adhesion to and invasion of A549 cells, evidenced by the intensified green fluorescence signals (PKH67-labeled EVs) colocalized with cell membranes and cytoplasm. Notably, this invasive phenotype followed a visible dose-dependent trend, correlating with the oxacillin concentration that the OS-200 strain was exposed to (Figure 10C).

#### 3.5.3 OI-EVs exerted proapoptotic effects on A549 cells

**Figure 11:**
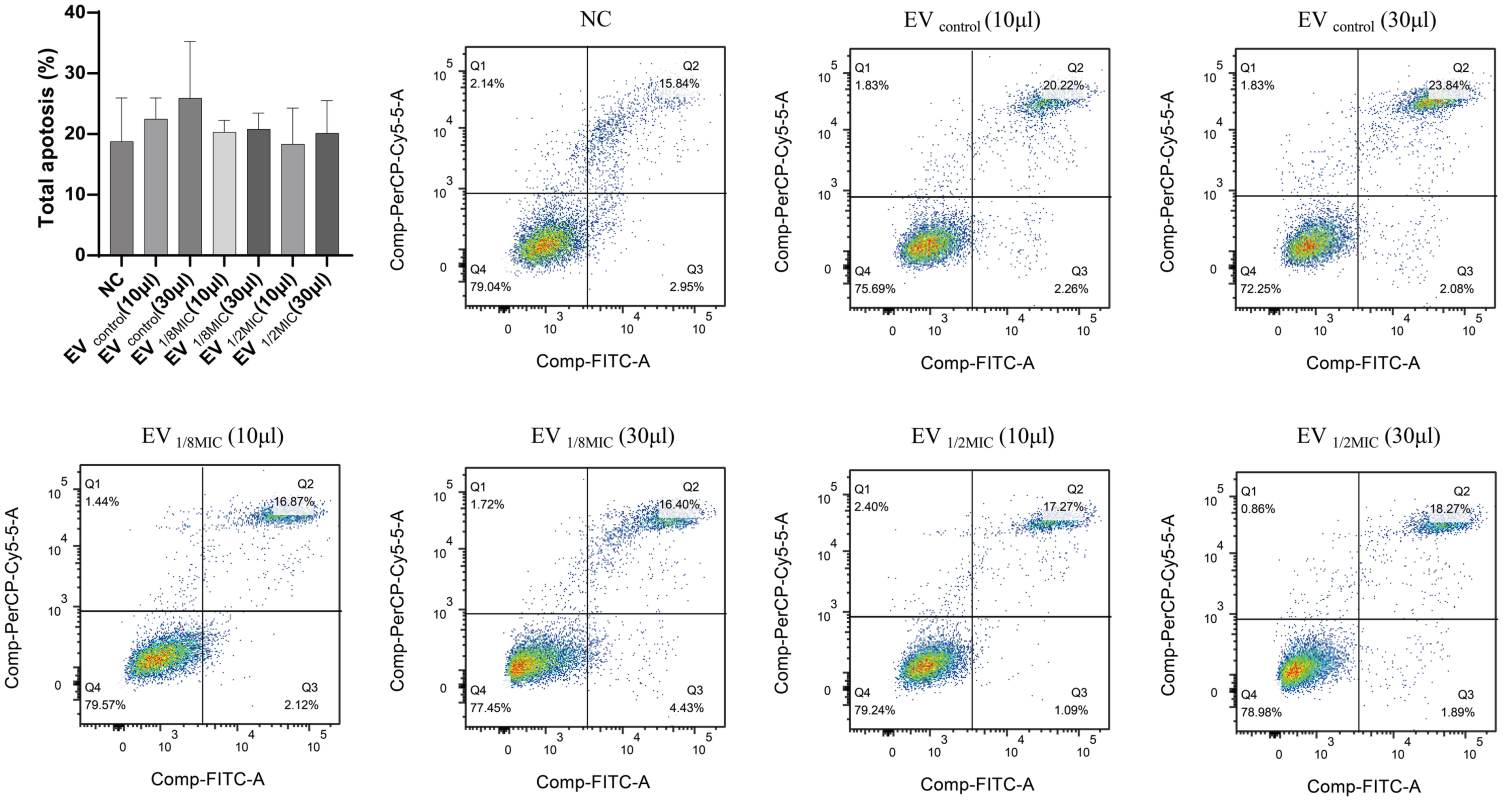
Pro-apoptotic effect of OI-EVs on A549 cells. The four flow cytometry quadrants represent distinct cellular states as follows: the lower left quadrant (FITC^−^/PI^−^) contains viable cells exhibiting no annexin V binding or PI uptake; the lower right quadrant (FITC^+^/PI^−^) represents early apoptotic cells characterized by annexin V-positive staining with intact cell membranes excluding PI; the upper right quadrant (FITC^+^/PI^+^) represents late apoptotic/necrotic cells demonstrating both annexin V binding and PI uptake due to membrane disruption; the upper left quadrant (FITC^−^/PI^+^) typically indicates mechanically damaged or necrotic cells showing PI uptake without annexin V binding. Data are presented as mean ± SEM and analyzed using Student’s *t-*test. ***p < 0.001, **p < 0.01, *p < 0.05.

Flow cytometry analysis revealed that all three EV preparations induced apoptosis in A549 cells to varying extents. Compared with the baseline apoptosis rate of 18.79% in the negative control group, treatment with 30 μL of EV_control_, EV_1/8MIC_, and EV_1/2MIC_ increased the apoptosis rate to 25.92, 20.83, and 20.16%, respectively. Notably, apoptosis rates in the high-volume groups (30-μl treatments) were consistently elevated relative to those in the low-volume groups (10-μl treatments), with a mean increase of 2.36% between the two groups (Figure 11).

### 3.6 OI-EVs boosted the production of inflammatory factors from macrophages

**Figure 12:**
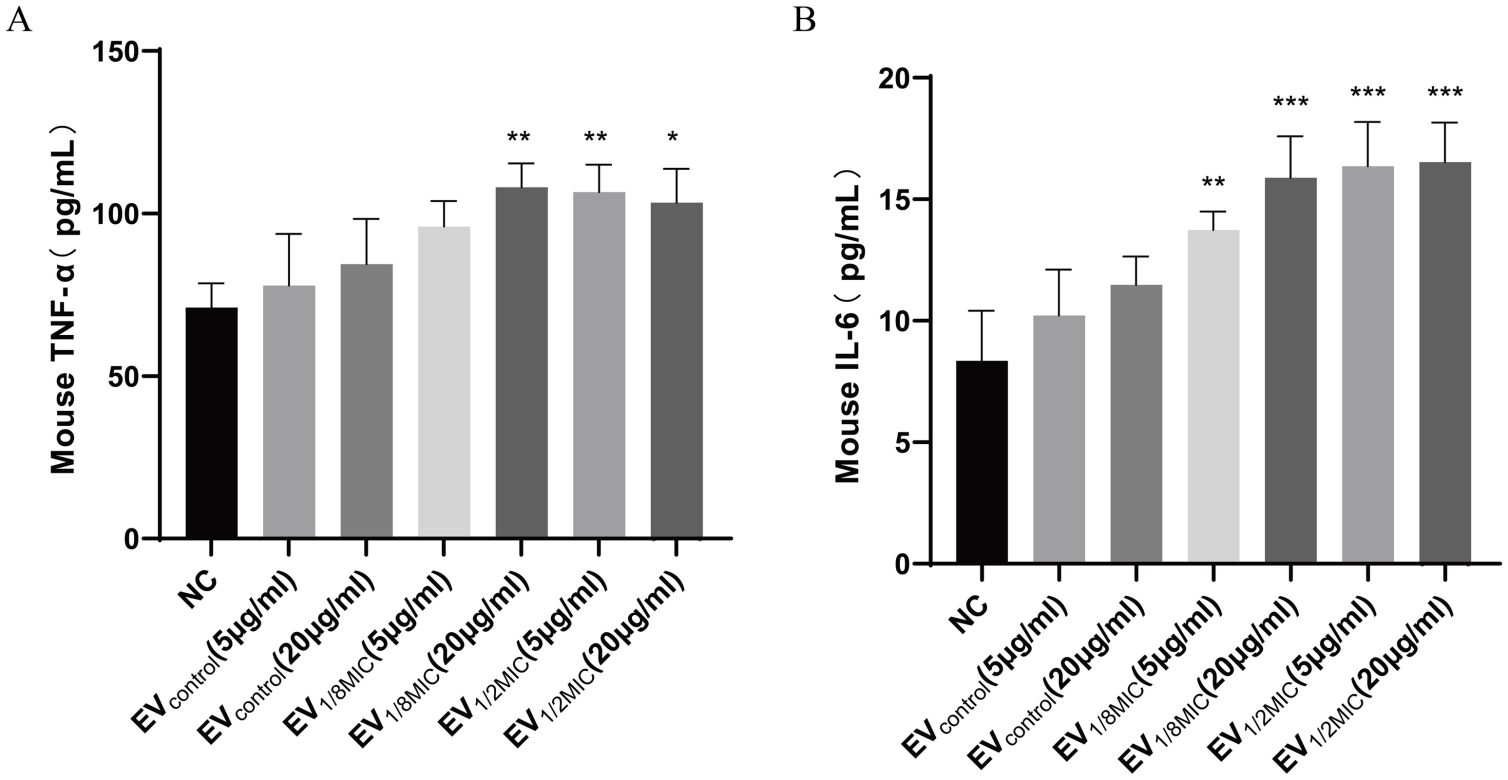
OI-EVs-mediated activation of macrophage inflammatory responses. **(A,B)** Two concentrations (5 μg/mL and 20 μg/mL) of the three EV preparations were coincubated with macrophages. The concentrations of TNF-α and IL-6 in the supernatants were determined using an ELISA kit. Data are presented as mean ± SEM and analyzed using Student’s *t-*test. ***p < 0.001, **p < 0.01, *p < 0.05.

This study evaluated the impact of OI-EVs on inflammatory cytokine release from macrophages using ELISA. The results demonstrated that OI-EVs induced macrophages to secrete elevated levels of inflammatory cytokines (TNF-*α* and IL-6) in a dose-dependent manner when exposed to sub-inhibitory concentrations of oxacillin. In particular, treatment with EV_1/2MIC_ increased the levels of TNF-α and IL-6 by 1.5- and 1.97-fold, respectively, compared with treatment with the negative control group (Figure 12).

## 4 Discussion

Antimicrobial agents are critical factors influencing the biological activities of *S. aureus*. They promote the secretion of virulence-associated proteins in free form, thereby affecting various pathogenic processes, including bacterial adhesion and invasion (Dumitrescu et al., 2011; Shang et al., 2019), biofilm formation (Kaplan et al., 2012), and the development of small-colony variants (SCVs) (Chen et al., 2021). Notably, both biofilm formation and SCV emergence are closely associated with the multidrug resistance of *S. aureus* (Guo et al., 2022). However, studies addressing the influence of antimicrobial agents on secretion and function of EV-associated proteins remain scarce. This study systematically investigated the impacts of sub-inhibitory concentrations of oxacillin on the secretion and functions of EVs from a clinical OS-MRSA isolate. This study reports various novel findings, including proteomic profile of EVs as well as its effects on host cells and the bacteria themselves.

A study in 2021 reported that the production of EVs by *S. aureus* was enhanced under various environmental stresses, including specific temperatures, oxidative stress, iron limitation, and sub-inhibitory concentrations of ethanol or antibiotics (Wang et al., 2021). A similar phenomenon was observed in our study—exposure to sub-inhibitory concentrations of oxacillin significantly increased the secretion and protein content of EVs produced by OS-MRSA. The results of differential proteomics analysis indicated that exposure to sub-inhibitory concentrations of oxacillin triggered the upregulation of various virulence-associated proteins in *S. aureus*, including virulence factors, adhesion-related proteins, toxins, and virulence regulators. Among the upregulated proteins, the expressions of PBP2, EmrB, Hlg, HlgB, QoxB, CtaB, and enolase increased in a concentration-dependent manner.

Under high-concentration antibiotic stress (1/2 MIC oxacillin), resistance mechanisms were robustly activated in *S. aureus*, coupled with remodeling of energy metabolism. The significant upregulation of two key *β*-lactam resistance-related proteins, PBP2 and BlaZ, indicated that the activities of cell wall synthesis and antibiotic hydrolysis were enhanced in response to antibiotic pressure. Concurrently, the pathways crucial for energy metabolism were activated, including heme metabolism (e.g., upregulated heme IX farnesyltransferase CtaB), respiratory chain function (e.g., upregulated quinol oxidase subunit QoxB), and glycolysis (e.g., enriched phosphofructokinase activity). This suggests metabolic rewiring, particularly an increased reliance on aerobic respiration, to meet the high energy demands of resistance mechanisms. Crucially, EV-associated proteins under high-concentration oxacillin stress primarily comprised toxins, such as β-channel-forming hemolysins (Hlg, HlgB) and pigment synthesis enzymes (CtaB and QoxB). This indicates that high-concentration antibiotic stress may have a more pronounced impact on the virulence of EVs produced by OS-MRSA. Notably, high-concentration antibiotic stress also led to the upregulation of virulence factors, such as cytolysin in the *S. aureus* infection pathway, implying a potential concurrent enhancement of resistance and virulence under intense antibiotic pressure.

By contrast, under low-concentration antibiotic stress (1/8 MIC oxacillin), *S. aureus* prioritized maintaining basal metabolic homeostasis. DEPs were significantly enriched in carbohydrate metabolism pathways (e.g., glycolysis, galactose metabolism) and fatty acid metabolism, reflecting optimization of carbon source utilization and energy conservation instead of the activation of energetically costly canonical resistance mechanisms. Proteomic analysis revealed that upon exposure to low concentration of oxacillin, EVs produced by *S. aureus* encapsulated proteins that primarily participate in detoxification processes, including oxidative stress responses (glucose-6-phosphate dehydrogenase and the heat shock proteins GroEL/GroES) and metal ion homeostasis (ferritin). Regarding antibiotic resistance mechanisms, only BlaZ was upregulated within the *β*-lactam resistance pathway, whereas cytolysin expression was downregulated. This suggests that under low-concentration antibiotic stress, *S. aureus* may rely more on the relatively “energy-efficient” strategy of enzymatic antibiotic degradation while suppressing certain virulence factors to potentially avoid triggering a premature robust host immune response. Furthermore, enrichment of DNA-binding transcription factor activity hints at a potential role for epigenetic regulation in long-term bacterial adaptation to low-concentration antibiotic stress.

Interestingly, the trend in virulence factor expression displayed a “biphasic effect” depending on the concentration of antibiotic exposure. The expression level of virulence factors, including Hlg, HlgB, β-channel-forming cytolysin, CymR, FemA, and MgrA, followed the order EV_1/2MIC_ > EV_control_ > EV_1/8MIC_ (Table 1). This clearly demonstrates that high antibiotic concentrations significantly promote the release of virulence factors, whereas low antibiotic concentrations exert an inhibitory effect. This observation aligns with the results of the hemolysis assay, demonstrating that sub-inhibitory antibiotic concentrations, particularly higher sub-inhibitory concentrations, can enhance virulence, potentially through the quorum sensing pathway. In line with these findings, the EV_1/2MIC_ vs. EV_1/8MIC_ comparison revealed concomitant enrichment in ferric iron binding and toxin activity (e.g., β-pore-forming cytolysin), further suggesting that disruption of metal ion homeostasis, particularly iron homeostasis, may be a key signal triggering virulence expression under high-concentration antibiotic stress.

GO enrichment analysis indicated that although the specific DEPs varied with the concentration of antibiotic exposure, their effects on EVs shared fundamental commonalities. Oxacillin exposure primarily targeted mechanisms governing the biogenesis and secretion of EVs and their functions as intercellular signaling vehicles, particularly in substance transport and metabolic regulation. Alteration in the protein composition of EVs, notably proteins affecting membrane architecture, transmembrane transport, and core metabolic processes, represents a universal cellular response pathway to antibiotic stress. These alterations may enable EVs to mediate intercellular metabolic coordination, stress signal propagation, or microenvironmental remodeling.

KEGG pathway analysis revealed that despite concentration-specific differences, core adaptive mechanisms were consistently evident in *S. aureus* exposed to antibiotic stress. The ABC transporter pathway was significantly enriched in all experimental groups, underscoring its fundamental role in antibiotic efflux, nutrient uptake, and environmental signal perception. This pathway forms a critical basis for bacterial multidrug resistance. Similarly, the quorum sensing pathway was significantly enriched in both the EV_1/2MIC_ vs. EV_control_ and EV_1/2MIC_ vs. EV_1/8MIC_ comparisons, indicating its pivotal role in coordinating the trade-off between resistance and virulence in *S. aureus* exposed to high-concentration antibiotic pressure.

Bacterial EVs can induce pathological changes in host cells via the transfer of pathogenic factors (Briaud and Carroll, 2020). A study conducted in 2016 indicated that variations in the proteome of *S. aureus* membrane vesicles could affect their toxicity to host cells (Sibbald et al., 2006; Jeon et al., 2016). Therefore, we sought to investigate how the proteomic changes in OI-EVs influenced their biological activity as well as the pathogenicity of the OS-200 strain. As a key pathogenic mechanism, the hemolytic ability of *S. aureus* is associated with several types of hemolysins, including free forms and those encapsulated in EVs, such as *α*, *β*, *γ*, and *δ* toxins. These toxins can damage red blood cells, disrupt platelet lysosomes, and induce local ischemia and necrosis (Jahn et al., 2022; Pivard et al., 2023; Jia et al., 2024). A study by Ohlsen et al. demonstrated that exposure to sub-inhibitory concentrations of β-lactam antibiotics significantly induced the expression of α-toxin (hla) in both methicillin-sensitive and methicillin-resistant *S. aureus* EVs, which enhances the α-toxin-dependent hemolytic activity of these EVs (Ohlsen et al., 1998). Furthermore, Thay et al. (2013) reported a strong association between α-toxin and EVs secreted by *S. aureus*. These EVs, containing biologically active α-toxin, exhibit cytotoxic effects on host cells, including erythrocyte lysis and apoptosis in epithelial cells (Thay et al., 2013). Collectively, these findings suggest that sub-inhibitory concentrations of β-lactam antibiotics may enhance the virulence of *S. aureus* by promoting α-toxin expression and its incorporation into EVs, thereby augmenting the cytotoxic potential of these vesicles. In this study, we found that EV_1/2MIC_ exhibited potent hemolytic activity against red blood cells. We believe that other hemolysins, in addition to α-hemolysin, are key factors affecting the hemolysis of red blood cells. For instance, proteomic analysis of OI-EVs in this study revealed a significant upregulation of γ-hemolysin (Hlg, HlgB), which is also considered an important effector protein promoting vesicular hemolytic activity. Notably, another unique finding of this study is the significant upregulation of the heme biosynthesis-related proteins CtaB and QoxB in OI-EVs. Heme biosynthesis is believed to primarily mediate the production of terminal oxidases in the bacterial respiratory chain (Ahmed et al., 2018). However, a study by Xu et al. revealed that the deletion of *ctaB* attenuates the hemolytic activity of *S. aureus* while enhancing pigment production during the stationary phase and the formation of quinolone-resistant viable cells (Xu et al., 2016). Furthermore, the two terminal oxidases also play an important role in the adaptation and pathogenicity of *S. aureus* during colonization (Hammer et al., 2013). These findings suggest that the virulence factors of *S. aureus* are intricately interrelated, and a specific pathogenic outcome may not be solely mediated by a single virulence factor.

The pathogenicity of *S. aureus* is closely linked to its ability to directly adhere to host cells or the extracellular matrix (Josse et al., 2017). Bacterial attachment is the initial step in host cell invasion and biofilm formation (Shang et al., 2019). This study demonstrated that OI-EVs enhance the invasive capacity and biofilm-forming ability of OS-MRSA. *S. aureus* invades host cells through a zipper-like mechanism, resembling professional phagocytosis, which requires bacterial surface proteins called adhesins or microbial surface components that recognize adhesive matrix molecules (Yang and Ji, 2014). Most *S. aureus* strains are believed to mediate host cell invasion via host integrin *α*5*β*1, a process facilitated by the binding of fibronectin-binding proteins A and B to fibronectin (Chen et al., 2021; Robertin et al., 2023). A study in 2011 demonstrated that exposure to sub-inhibitory concentrations of oxacillin significantly increased the transcription of *S. aureus* fnbA/B, thereby enhancing bacterial attachment to host cells (Rasigade et al., 2011). In our proteomics analysis, we found that several other attachment-related proteins were significantly upregulated under sub-inhibitory concentrations of oxacillin, such as Eno, SrtA, and DegP/HtrA. Therefore, we propose that stimulation with β-lactam antibiotics primarily induces the secretion of adhesion-related proteins in their free form or encapsulated within EVs, thereby promoting bacterial attachment and invasion of host cells. Another study showed that cefotaxime can stimulate OS-MRSA to produce more EVs, which form bridges between bacteria, thereby increasing surface hydrophobicity and eventually inducing bacterial aggregation to form biofilms (He et al., 2019). On the basis of these findings, we hypothesize that the mechanism through which OI-EVs enhance biofilm formation in OS-MRSA may be more complex, and further research is required to fully elucidate the specific role of vesicles in this process.

Another crucial finding of this study is that OI-EVs can induce macrophages to release the inflammatory cytokines (TNF-α and IL-6) (Figure 12) in a concentration-dependent manner. Wang et al. demonstrated that EVs from *S. aureus* can activate key signaling pathways associated with inflammation and intercellular communication, particularly the nuclear factor-κB and mitogen-activated protein kinase pathways, thereby enhancing the host immune responses (Wang et al., 2023). Another study reported that sub-inhibitory concentrations of *β*-lactam antibiotics could upregulate lipoprotein genes (*lpl*, *sa2275*–*sa2273*) in MRSA strains (Shang et al., 2019). These lipoproteins, in turn, activate macrophages via Toll-like receptor 2-dependent pathways, leading to the production of proinflammatory cytokines and the amplification of host inflammatory responses (Haddadin et al., 2010). Similarly, our study revealed that exposure to sub-inhibitory concentrations of oxacillin increased the content of lipoproteins, such as Lpl9, GmpC, SAXG_01787, and SFAG_00213, in OS-200 EVs. We hypothesize that the upregulation of these lipoproteins may play a critical role in stimulating macrophages to produce more inflammatory cytokines in response to OI-EVs.

An important issue that remains to be addressed in this study is the controversial effect of OI-EVs on host cell proliferation and apoptosis. Several studies have suggested that *S. aureus* EVs can induce cell apoptosis; however, the underlying mechanisms remain unclear and debatable. Wang et al. proposed that *S. aureus* EVs can target the mitochondria of MAC-T cells, inducing the production of mitochondrial reactive oxygen species and superoxide radicals, which leads to mitochondrial dysfunction and, consequently, apoptosis (Wang et al., 2023). Additionally, another study suggested that *S. aureus* EVs could inhibit epithelial cell proliferation and induce apoptosis by upregulating apoptotic genes, such as *BAK1* and *BAG3* (Chen et al., 2022). Similarly, our study showed that EVs derived from OS-200, under both baseline and sub-inhibitory oxacillin conditions, inhibited A549 cell proliferation and induced apoptosis in a volume-dependent manner. However, unexpectedly, the apoptotic effect induced by OI-EVs was lower than that induced by EV_control_, prompting further investigation. Proteomic analysis of OI-EVs revealed a downregulation of several proteins associated with *S. aureus* colonization, infection, and secretion, including Hld, PSM, EsaA, IsaA, AmiD, and MraZ. Notably, Hld, EsaA, and IsaA showed a concentration-dependent downregulation.

A major limitation of this study is that it focuses on just one OS-MRSA strain, the heterogeneity of which may influence the composition and functions of EVs. Future studies should focus on more strains, especially those with classical phenotypic resistance to oxacillin and those resistant to other types of β-lactams.

## 5 Conclusion

In conclusion, exposure to antimicrobial agents at sub-inhibitory concentrations can influence the secretion efficiency and proteomic profile of EVs produced by OS-MRSA strains, eventually modulating the pathogenicity and antibiotic resistance of OS-MRSA. The findings of this study contribute to a better understanding of how sub-inhibitory antibiotic stress affects the secretion of virulence factors through vesicles in OS-MRSA strains, providing new insights into the prevention and treatment of OS-MRSA-related infections.

## Data Availability

The original contributions presented in the study are publicly available. This data can be found here: https://proteomecentral.proteomexchange.org/cgi/GetDataset?ID=PXD066440 (accession number: PXD066440). All other data supporting the findings of this study are available in the supplementary files of this article.
