# Supplementary material for: Sub-inhibitory concentrations of oxacillin modulate biogenesis and function of extracellular vesicles secreted by oxacillin-sensitive methicillin-resistant Staphylococcus aureus
Source: Front Microbiol. 2025 Aug 4;16:1616536. doi: 10.3389/fmicb.2025.1616536 (PMC12358487; doi:10.3389/fmicb.2025.1616536)

**Additional file 3**

The extracellular vesicles (EVs) from the three groups exhibited differential hemolytic activity on red blood cells. EVs derived from the EV_control_ and EV_1/8 MIC_ groups showed minimal hemolytic activity, comparable to that of the blank control group treated with PBS alone. Consequently, the blood agar plates from these three groups displayed similar coloration. In contrast, EVs from the EV_1/2 MIC_ group demonstrated markedly enhanced hemolytic activity, as indicated by the significantly darkened appearance of the corresponding blood agar plate.


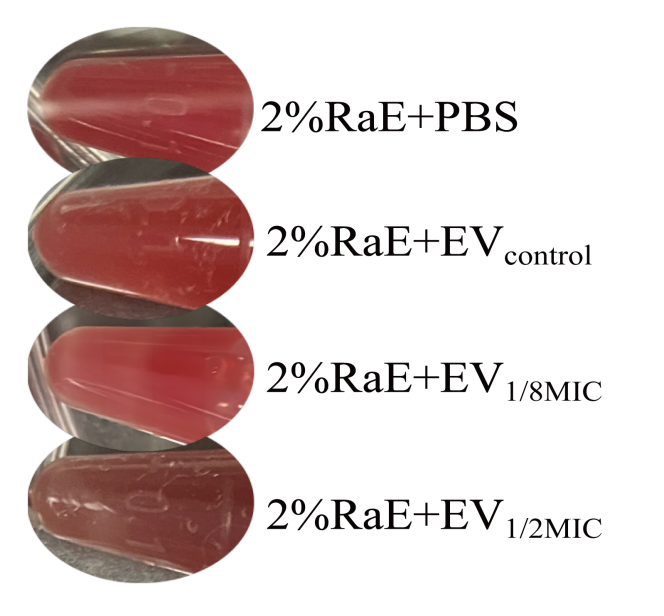

Supplement: Supplementary file 1 [file Data_Sheet_1.zip › Additional file 3.docx]
