# Supplementary material for: Sub-inhibitory concentrations of oxacillin modulate biogenesis and function of extracellular vesicles secreted by oxacillin-sensitive methicillin-resistant Staphylococcus aureus
Source: Front Microbiol. 2025 Aug 4;16:1616536. doi: 10.3389/fmicb.2025.1616536 (PMC12358487; doi:10.3389/fmicb.2025.1616536)

**Additional file 1**

Identification results of the OS-200 clinical isolate using matrix-assisted laser desorption/ionization time-of-flight mass spectrometry (MALDI-TOF MS).


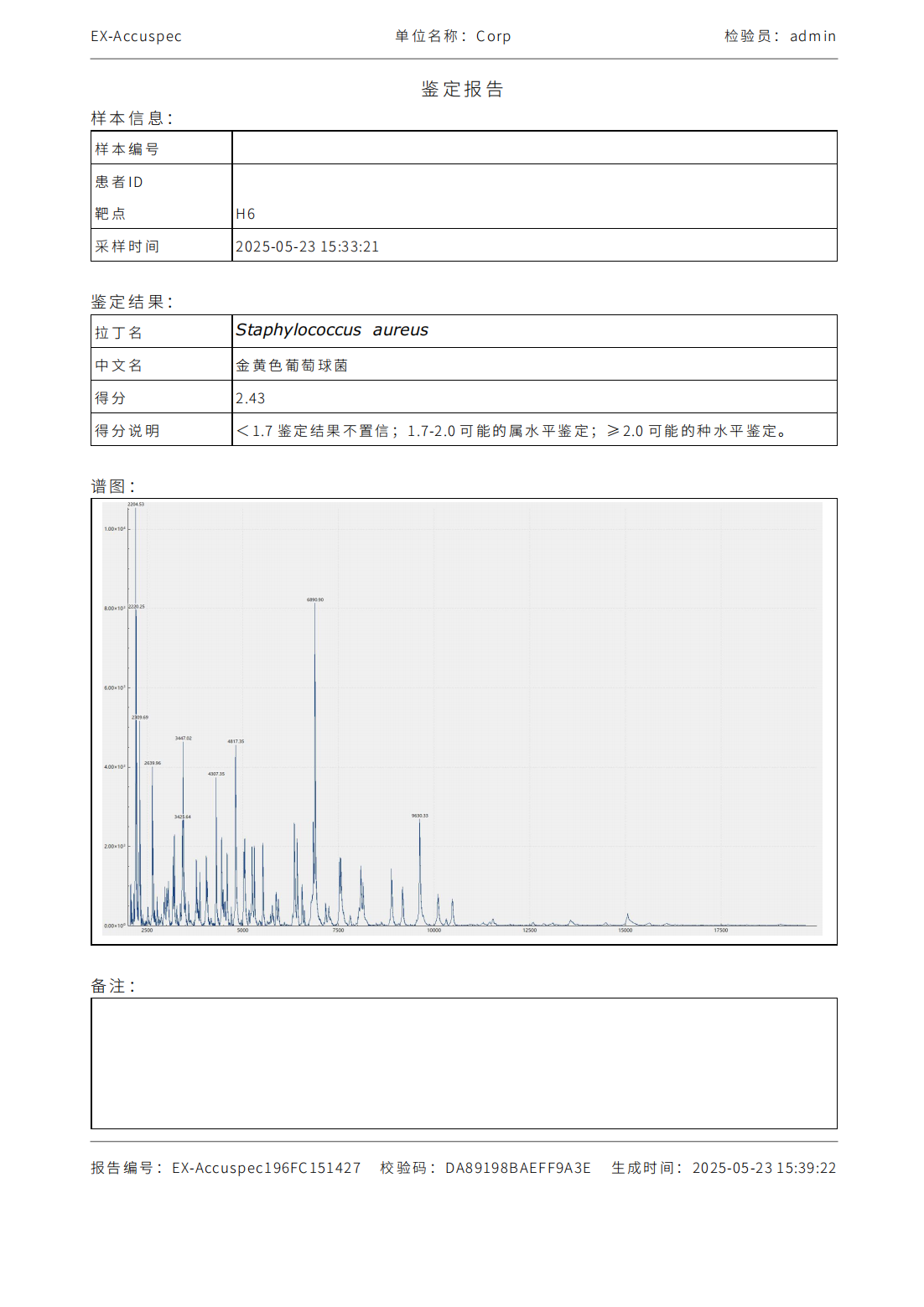

Supplement: Supplementary file 1 [file Data_Sheet_1.zip › Additional file 1.docx]
