# Supplementary material for: Sub-inhibitory concentrations of oxacillin modulate biogenesis and function of extracellular vesicles secreted by oxacillin-sensitive methicillin-resistant Staphylococcus aureus
Source: Front Microbiol. 2025 Aug 4;16:1616536. doi: 10.3389/fmicb.2025.1616536 (PMC12358487; doi:10.3389/fmicb.2025.1616536)
Supplement: Supplementary file 1 [file Data_Sheet_1.zip › Additional file 2.docx]

**Additional file 2**

A comparison of three EVs groups (EV _control_, EV_1/8MIC_ and EV_1/2MIC_) proteome.
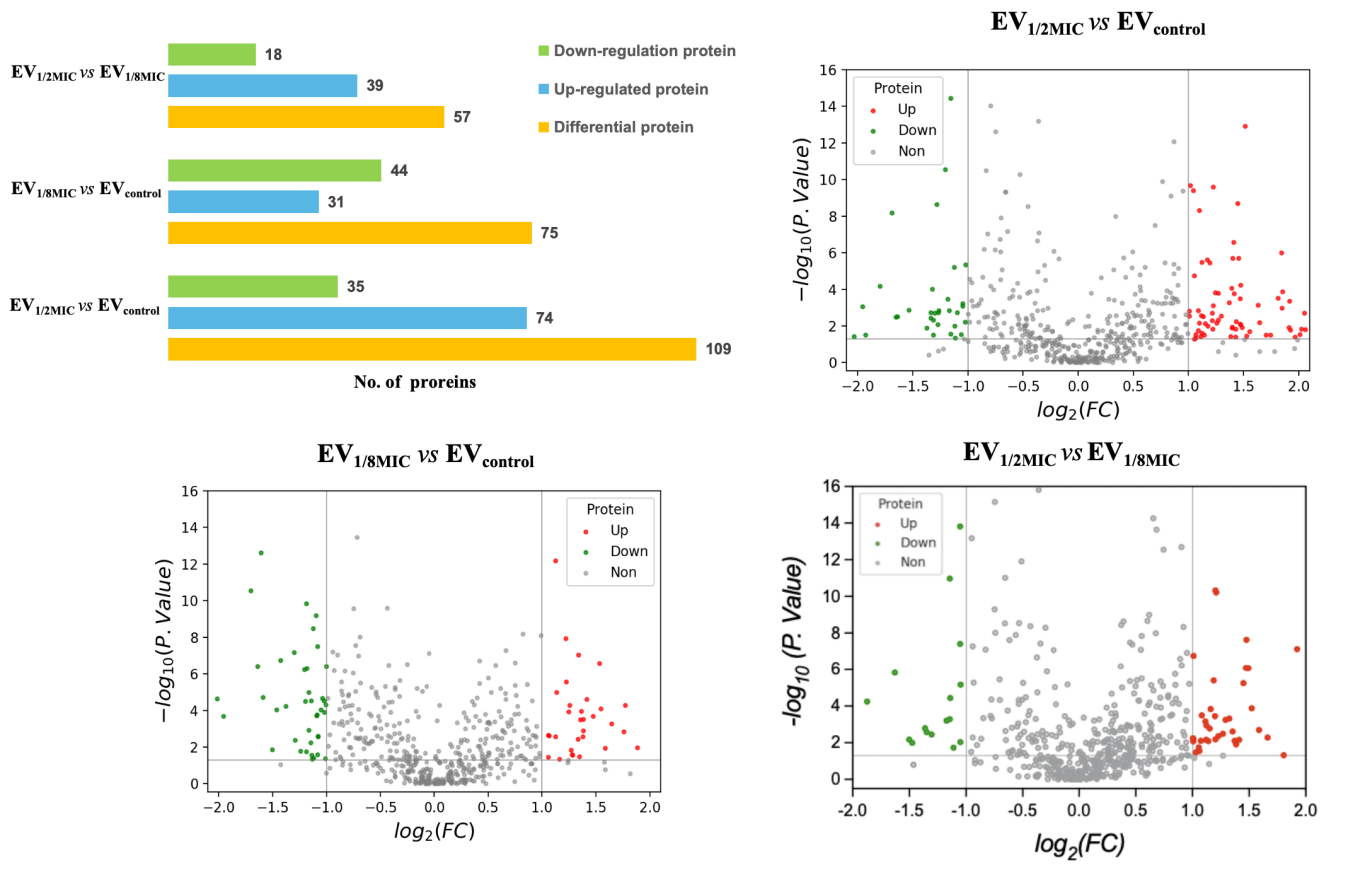


1. Differential protein numbers between three sets of OS-200 EVs. The analysis was performed by ANOVA analysis of variance with screening *p* <0.05 and greater than 2-fold difference as differential proteins. (B) Volcano plot of the three groups of OS-200 EVs. Red dots represent significantly up-regulated differential proteins, green dots represent significantly down-regulated differential proteins and grey dots represent non-significantly differential proteins. The abscissa is the value of the difference and the ordinate is P-value taking the value of -log10.
